# Revealing Putative Causal Genes by Establishing the Causality Between Different Lymphomas and Immune Cells

**DOI:** 10.1111/jcmm.70535

**Published:** 2025-05-13

**Authors:** Jingxuan Lian, Xinghong Zhang, Wenjie Chen, Zheshen Lin, Ming Lu, Rong Liang

**Affiliations:** ^1^ Department of Hematology, Xijing Hospital Air Force Medical University Xi'an Shaanxi China

**Keywords:** genome wide association studies, immune cells, lymphoma, mendelian randomization, single‐nucleotide polymorphisms, summary data‐based mendelian randomization, the tumour immune microenvironment

## Abstract

The tumour immune microenvironment (TIME) is critical for lymphoma progression and therapy resistance, yet causal relationships between specific immune cell types and lymphoma subtypes remain poorly defined. In this study, using bidirectional Mendelian randomization (MR), genetic correlation (LDSC), and expression‐QTL integration (SMR), we systematically evaluated causal relationships and genetic correlation between immune cells and various lymphomas. Additionally, we utilised the Mendelian randomization‐based method of summary data‐based MR (SMR), which incorporated genome‐wide association studies (GWAS) and expression quantitative trait loci (eQTL) data from immune cells to identify genes associated with lymphoma. Furthermore, colocalization analysis and genetic correlation analysis were conducted for further validation of our findings. The two‐sample mendelian randomization approach was employed to identify the immune cell types that exhibit a causal relationship with different lymphomas. Additionally, the genetic correlation between these immune cells and lymphomas was further analysed using the linked disequilibrium score regression method, thereby enhancing the reliability of our findings. The SMR and colocalisation analyses revealed several genes associated with these immune cells, thereby providing additional support for their putative role in the pathogenesis of lymphoma. Our study elucidates the intricate interplay between immune cells by employing genetic methodologies, thus suggesting novel therapeutic candidates that warrant experimental validation and risk predictors in different subtypes of lymphoma treatments.

**Abbreviations**
BH: Benjamini‐Hochberg
DLBCL: diffuse large B‐cell lymphoma
eQTL: expression quantitative trait loci
GWAS: genome‐wide association studies
IVs: instrumental variables
IVW: inverse‐variance weighted
LD: linkage disequilibrium
LDSC: linked disequilibrium score regression
MDSCs: myeloid‐derived suppressor cells
MR: Mendelian randomization
NHL: non‐Hodgkin lymphoma
PP.H4: posterior probability of shared causal variant
SMR: summary data‐based Mendelian randomization
SNPs: single‐nucleotide polymorphisms
TIME: tumour immune microenvironment
TME: tumour microenvironment

## 1 Introduction

Lymphoma, comprising more than 90 subtypes of lymphocyte neoplasms, is conventionally classified broadly as non‐Hodgkin or Hodgkin lymphoma. Each year, approximately 82,000 new cases of lymphoma are diagnosed in the United States. Lymphoma accounts for ~4% of all cancer deaths globally, with subtypes like DLBCL and Hodgkin lymphoma showing distinct immune microenvironment signatures [1]. The prevailing belief is that malignant lymphocytes in lymphoma primarily recruit and sustain a microenvironment consisting of other immune cells as well as stromal elements, which facilitate the promotion of malignant cell growth and survival [2]. The biology and clinical behaviour of lymphoma are not solely determined by the intrinsic characteristics of tumour cells, but are also significantly influenced by their dynamic interaction with the non‐malignant microenvironment [3]. The tumour immune microenvironment (TIME) is a recently proposed concept that has been demonstrated to exhibit a robust association with the clinical outcomes of cancer patients [4]. The tumour microenvironment (TME) predominantly consists of a diverse array of immune cell populations, encompassing both innate and adaptive immune cells such as myeloid cells and lymphocytes [5, 6]. It is noteworthy that TIME also plays a pivotal role in determining the immune response state within the tumour microenvironment (TME), which predominantly relies on the composition and activity of infiltrating immune cells, alongside various influencing factors such as cell surface expression of immune checkpoint molecules and alterations in associated extracellular matrix [5]. The composition of immune cells within the tumour immune microenvironment (TIME) exhibits inter‐tumoral variability and demonstrates significant associations with clinical outcomes across diverse cancer types [7].

The immune microenvironment plays a crucial role in the pathogenesis, disease progression, and therapy resistance of lymphomas. In diffuse large B‐cell lymphoma (DLBCL), the composition of various immune effectors and cells can serve as prognostic biomarkers and independent indicators for different immunotherapies [8, 9]. The presence of myeloid‐derived suppressor cells (MDSCs) has been observed in the peripheral blood of patients with Hodgkin and Non‐Hodgkin lymphoma (NHL), demonstrating a positive correlation with disease aggressiveness and significant prognostic value [10, 11]. The tumour microenvironment (TME) is widely recognised to play a crucial role in various processes, including lymphoma progression, treatment response, drug resistance, and prognosis. Targeting components of the TME holds promise for uncovering novel insights into precise lymphoma management. Furthermore, by employing Mendelian randomization in epidemiological etiological inference, we establish a methodological foundation to elucidate potential causal relationships between distinct immune cells and lymphoma as well as its subtypes. In this study, To bridge the gap between observational and causal relationships, we applied genetic tools to identify immune cell drivers of lymphoma development. we conducted a comprehensive bidirectional Mendelian randomization analysis to elucidate the causal associations between diverse immune cell types and lymphoma along with its different subtypes.

## 2 Methods

### 2.1 Study Design Description

**FIGURE 1:**
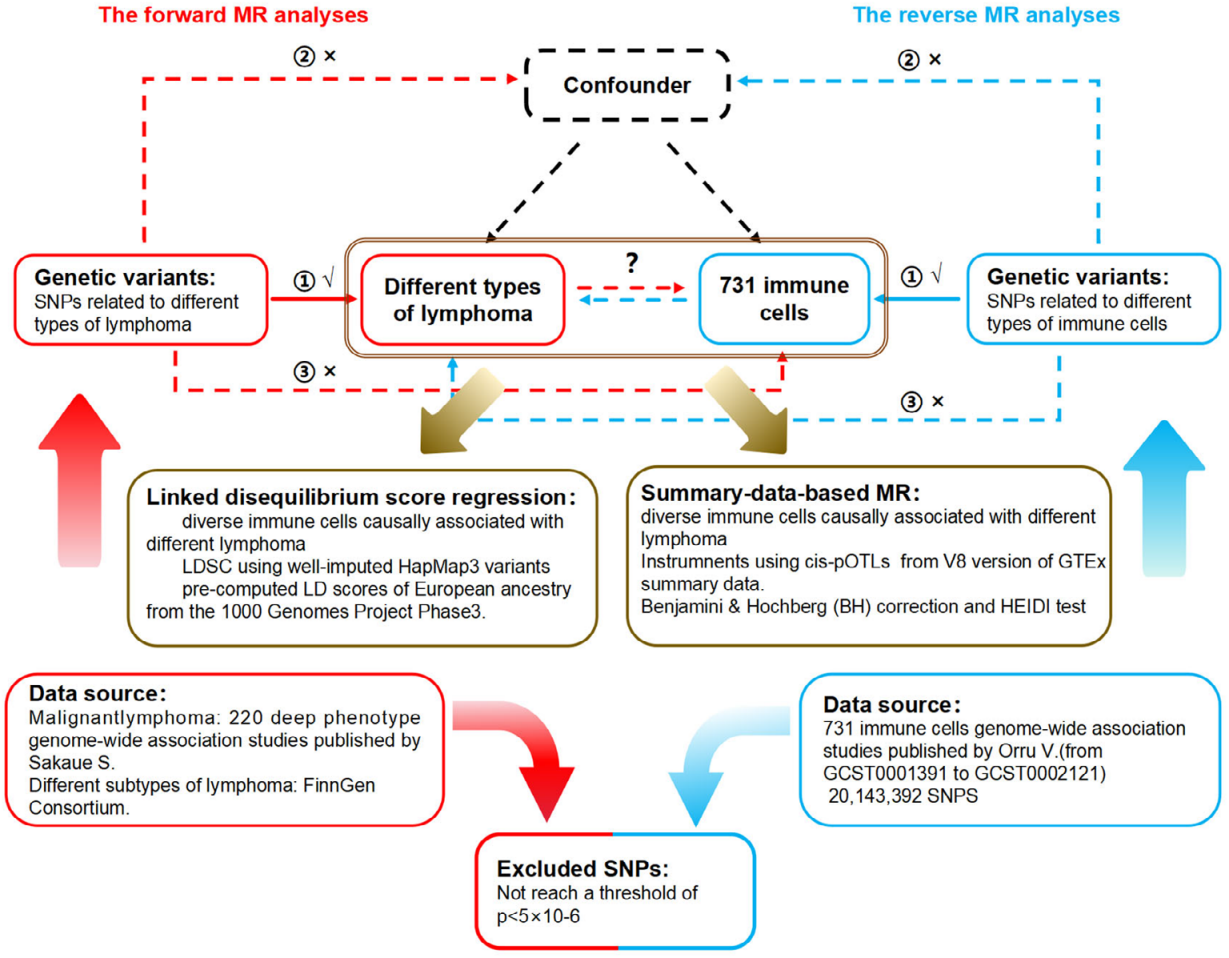
Description of the study design in this bidirectional MR study. Sketch of the study design. The red represented the forward MR analyses, with 731 immune cells as exposure and lymphoma and its different subtypes as the outcome. The blue represented the reverse MR analyses, with lymphoma and its different subtypes as exposure and 731 immune cells as the outcome. The immune cells previously examined for their causal association with various lymphomas were further subjected to SMR and LDSC analysis, as suggested by Brown. MR, Mendelian randomization; SNPs, single‐nucleotide polymorphisms.

The bidirectional Mendelian randomization (MR) design, as depicted in Figure 1, provides a concise overview of the investigation into the association between 731 immune cell types and lymphomas, encompassing their diverse subtypes. First, we used GWAS data to perform bidirectional MR analyses between immune cells and lymphoma subtypes. Subsequently, Linkage disequilibrium score regression (LDSC) was utilised to further investigate the genetic correlation of immune cells causally associated with two or more lymphomas, as indicated by the previous MR Results. Second, we regarded lymphoma and its subtypes as exposures with subsequent effects on the 731 immune cell types. Third, by employing SMR analysis, we performed a joint analysis of GWAS and eQTL aggregate statistics to identify functionally relevant genes at the loci identified in GWAS.

### 2.2 Data Sources for Lymphoma and Its Different Subtypes of GWAS

Regarding the diverse types of lymphoma GWAS database utilised in this study, it was primarily sourced from FinnGen—an extensive project involving genetic data collection and analysis from over 500,000 participants within the Finnish Biobank [12]. Our study aimed to investigate the causal relationship between six distinct subtypes of lymphoma, including DLBCL, follicular lymphoma, and non‐follicular lymphoma, and a total of 731 immune cells.

### 2.3 Immunity‐Wide GWAS Data Sources

The aggregated GWAS statistics for all 731 immune traits can be accessed publicly through the GWAS catalogue, spanning from GCST0001391 to GCST0002121 [13]. The study encompassed a total of 731 immunophenotypes. Specifically, the B cells, cDCs, mature T cells, monocytes, bone marrow cells, TBNK (T cells, B cells, natural killer cells), and Treg cells were characterised by MFI, AC, and RC features. Conversely, the cDCs and TBNK panels constituted the MP features. The genome‐wide association analysis of immune traits employed data from a non‐overlapping cohort of 3757 Europeans. A comprehensive set of genetic variants comprising 20,143,392 SNPs and 16,888,858 indels was examined using either high‐density genotyping arrays or a reference panel based on Sardinian sequences and tested for association after adjusting for covariates (i.e., sex, age and age^2^) [14].

### 2.4 Genetic Instrumental Variants (IVs) Selection

In strict accordance with the three core assumptions of MR Study designs (1) there is a strong association between instrumental variables (IVs) and exposure factors; (2) IVs are independent of confounding factors in the expose‐outcome relationship; (3) Genetic variation can only affect results through exposure, and not through other ways [15]. To identify instrumental variables for various exposure factors, single nucleotide polymorphisms (SNPs) at the genome‐wide significance threshold (*p* < 5 × 10^−6^) were extracted from the immune traits genome‐wide association study (GWAS). Linkage disequilibrium (LD), with an *r* ^2^ value of 0.01 and a clumping distance of 500, was calculated using the reference panel provided by the 1000 Genomes Project. Additionally, SNPs not present in the outcome GWAS were removed; proxy SNPs were not utilised in this study. The remaining SNPs were used for Mendelian randomization analysis. F statistics were computed to assess the strength of genetic instrumental variables, considering only those SNPs with an F statistic > 10 as non‐weak instruments.

### 2.5 Statistical Analysis

The main analyses involved three stages: two‐sample Mendelian analysis, primary SMR analyses, and colocalization analyses. The data were harmonised to exclude SNPs with ambiguous alleles and palindromic SNPs. The primary MR analyses were conducted using the multiplicative random‐effects inverse‐variance weighted (IVW) method, which provides precise estimates under the assumption of all SNPs being valid instruments. Estimates from different sources were combined using fixed‐effects meta‐analysis, and heterogeneity across associations was assessed using the *I* ^2^ statistic for different data sources and Cochran's *Q* value for SNP estimates within each association. The *I* ^2^ statistic was calculated to assess the heterogeneity of each outcome from different data sources, and the *I* ^2^ values < 25%, 25%–75%, and > 75% were considered to indicate low, moderate, and high heterogeneity, respectively. Sensitivity analyses including weighted median, MR‐Egger, and MR pleiotropy residual sum and outlier (MR‐PRESSO) analyses were performed to detect potential unbalanced pleiotropy (horizontal pleiotropy) and examine consistency of associations [16]. Additionally, MR‐PRESSO can effectively correct the outliers in the instrumental variables (IVs) and provide an estimate that is consistent with IVW after removing these outliers, as indicated by a significant *p*‐value < 0.05 demonstrating the presence of directional pleiotropy [17]. Instrument strength was quantified by estimating the F‐statistic, where an F‐statistic > 10 indicated a sufficiently strong instrument. Power analysis was conducted using an online tool. To account for multiple testing, the Benjamini‐Hochberg correction controlling false discovery rate was applied. Associations with nominal *p*‐values < 0.05 and Benjamini‐Hochberg adjusted *p*‐values > 0.05 and < 0.1 were considered suggestive, while those with Benjamini‐Hochberg adjusted *p*‐values < 0.05 were deemed significant.

### 2.6 Summary‐Data‐Based MR (SMR)

The Summary‐data‐based Mendelian randomization (SMR) method was employed to generate effect estimates when utilising expression quantitative trait loci (eQTLs) as instrumental variables, enabling the investigation of the association between gene expression levels and outcomes of interest using summary‐level data from genome‐wide association studies (GWAS) and eQTL studies. Allele harmonisation and analysis were conducted using version 1.03 of the SMR software. Detailed information regarding the SMR method has been previously reported [18]. The associations with HEIDI test *p* < 0.05 may be ascribed to linkage rather than pleiotropy, where the same variant independently regulates both outcomes and exposures; thus, such associations should be excluded from the analysis. In SMR analysis, cis‐eQTL genetic variation is used as an instrumental variable (IVs) of gene expression. We used lymphocyte eQTL data for SMR analysis. eQTL data comes from the V8 version of GTEx summary data. Detailed information on sample collection and processing is provided elsewhere. EQTL data can be downloaded from https://cnsgenomics.com/data/SMR/#eQTLsummarydata.

### 2.7 Colocalization Analysis

The colocalization approach serves as a means to evaluate the presence of shared causal variations between two features within a given genomic region. To enhance the precision of our findings, we conducted an additional Bayesian test for colocalization of two traits using the coloc R package (https://chr1swallace.github.io/coloc/, version 5.1.0) in order to estimate the posterior probability of shared variants. The basic hypothesis for colocalization in the same genomic location is: H0: neither trait has a causal genetic variant; H1: only trait 1 has a causal genetic variant; H2: only trait 2 has a causal genetic variant; H3: both traits have a causal genetic variant, but not the same variant; H4: both traits share the same causal variant. For each leading SNP in the gastrointestinal disease GWAS database under investigation, all SNPs within a 100 kb range upstream and downstream from the leading SNP were retrieved for co‐localization analysis, aiming to assess the posterior probability of H4 (PP.H4). A PP.H4 value greater than 0.75 was considered a robust threshold indicating evidence supporting co‐localization between GWAS and QTL associations.

### 2.8 Linked Disequilibrium Score Regression

We used linked disequilibrium score regression (LDSC) and assessed genome‐wide genetic associations between different immune cells that were causally associated with six types of lymphoma. Genetic correlation analyses were performed according to the standard analysis process of LDSC. We performed LDSC using well‐imputed HapMap3 variants and pre‐computed LD scores of European ancestry from the 1000 Genomes Project Phase3. We did not constrain the intercepts in the LDSC analysis, which could not only account for residual confounding but also indicate whether there was potential sample overlap between two GWAS studies.

## 3 Results

### 3.1 Analysis and Comparison of Causal Immune Cells in Different Types of Lymphoma

**FIGURE 2:**
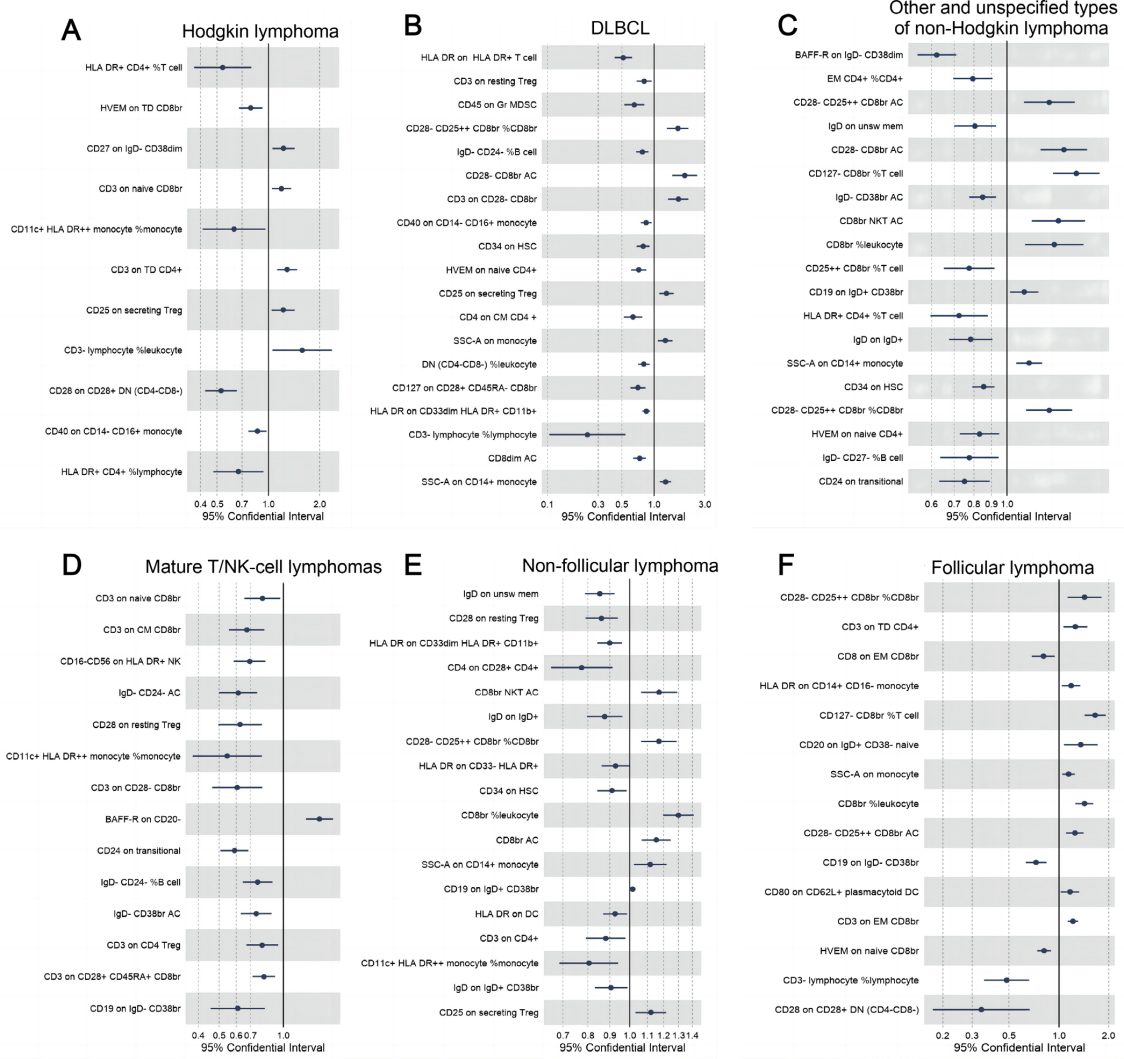
Immune cells exhibiting a causal association with various types of lymphomas by IVW. (A) The immune cells causally associated with Hodgkin's lymphoma; (B) DLBCL; (C) other and unspecified non‐Hodgkin lymphoma; (D) mature T/NK‐cell lymphomas; (E) non‐follicular lymphoma; (F) follicular lymphoma. Error bars: 95% confidence intervals of IVW estimates.

After conducting MR analysis and applying BH correction, we identified a series of distinct immune cell types that exhibited causal relationships with six lymphomas (Figure 2A–F). Table S1 provides detailed information on these specific immune cell types. We identified 11 immune cells linked to Hodgkin lymphoma, 19 to DLBCL, 19 to other NHL, 14 to T/NK‐cell lymphoma, 18 to non‐follicular lymphoma, and 15 to follicular lymphoma. Moreover, both the MR‐Egger truncation test and the MR‐PRESSO global test effectively ruled out pleiotropy at the level of causal immune cell associations across different lymphomas. Sensitivity analyses further confirmed the robustness of our observed causal relationships. Comprehensive information can be found in Table S2. The heterogeneity analysis revealed that the majority of results exhibited homogeneity, while those with heterogeneity demonstrated only mild levels (*I* ^2^ < 25%). A small subset of findings displayed moderate heterogeneity (25% < *I* ^2^ < 75%), and Significant heterogeneity was observed in a limited number of findings (mature T/NK‐cell lymphomas: CD3 on CD4, *I* ^2^ = 75.7%; non‐follicular lymphoma: HLA DR on CD33dim HLA DR^+^ CD11b^+^, *I* ^2^ = 75.7%, IgD on IgD^+^CD38br, *I* ^2^ = 76%), thus indicating the robustness of our analytical findings. Additionally, to investigate whether there existed a causal relationship between lymphomas and the aforementioned immune cell types, we conducted inverse Mendelian randomization analysis as an extension to our study. The corresponding results are provided in Table S3. Our analysis revealed a unidirectional association between these specific immune cell types and lymphoma rather than a bidirectional relationship.

### 3.2 Analysis and Comparison of Causal Immune Cells in Different Types of Lymphoma

**FIGURE 3:**
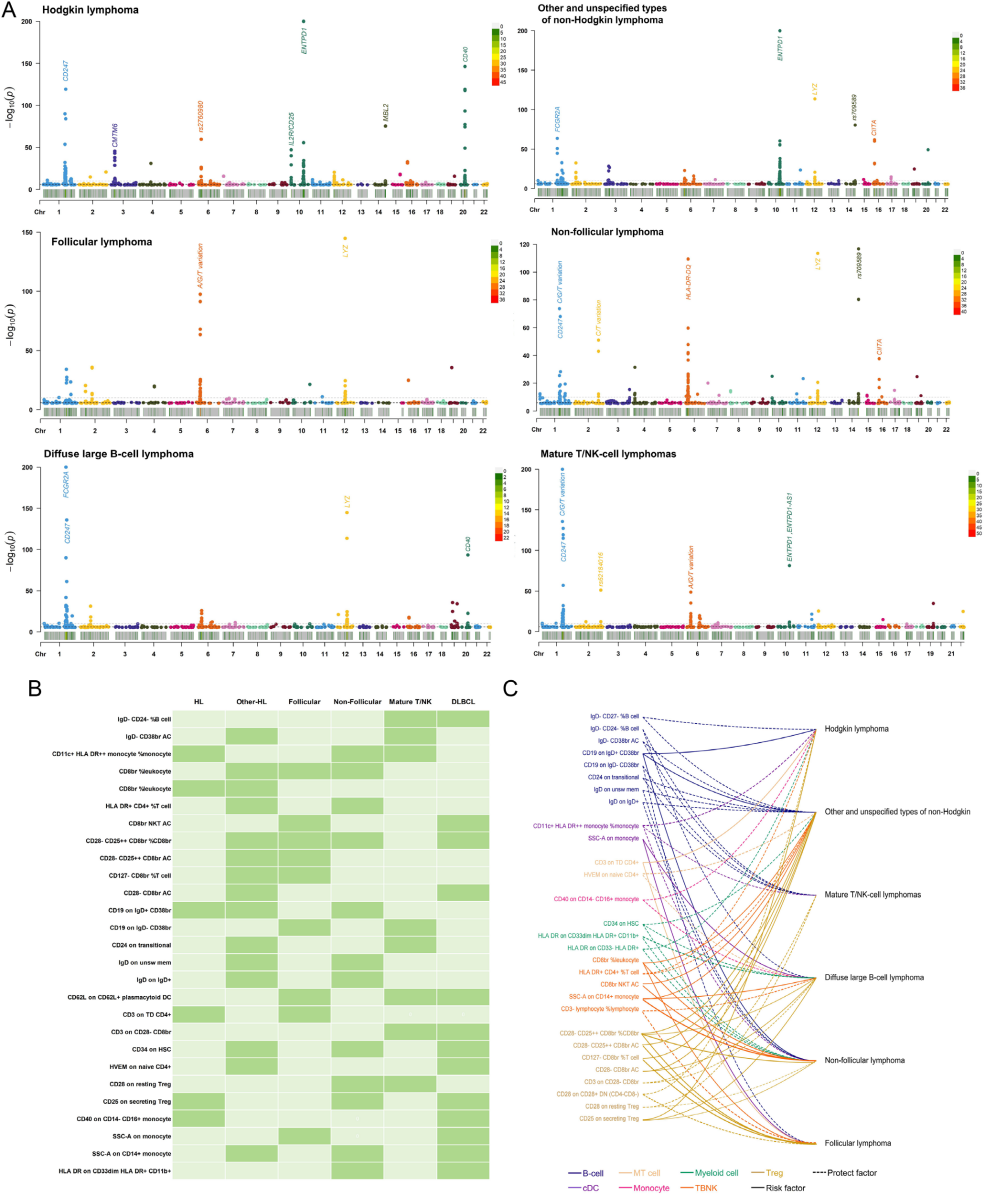
Analysis and comparison of causal immune cells. (A) Summary of the association between immune cells and gene loci with different subtypes of lymphoma; (B) Overlap of different causal immune cells in six lymphomas; (C) Direction of action of different immune cells on six lymphomas. Dotted lines are protective factors, solid lines are risk factors, and different colours represent different cell panels. HL, Hodgkin lymphoma; Other‐HL, other and unspecified types of non‐Hodgkin lymphoma.

To further explore the causal relationship between immune cells and various subtypes of lymphoma at the genetic level, we conducted an architectural analysis and employed a Manhattan plot (Figure 3A) to identify effector genes. Our findings exhibited significant correlations in Hodgkin's lymphoma between CD247, CMTM6, CD25, ENTPD1, MBL2, and CD40. In non‐Hodgkin lymphoma, including unspecified types, FCGR2A, ENTPD1, LYZ, CIITA, and rs709589 were found to be significantly associated. Notably, LYZ displayed a pronounced association specifically with follicular lymphoma but was also observed in non‐follicular lymphoma. Additionally, robust associations of CD247, HLADR‐DQ, and CIITA were evident. Furthermore, in DLBCL cases, CD247, FCGR2A, LYZ, and CD40 demonstrated significant associations. In mature T/NK cell lymphomas, a significant association was observed between CD247 and ENTPD1. Figure 3B highlights the co‐occurrence of immune cells that are causally linked to different types of lymphoma. A total of 28 immune cells were identified as being causally associated with two or more distinct lymphomas. Among these, 20 immune cells were associated with two lymphomas, seven immune cells were associated with three lymphomas, and notably, one immune cell was implicated in four lymphoma subtypes (including unspecified non‐Hodgkin lymphoma, DLBCL, follicular lymphoma, and non‐follicular lymphoma). Figure 3C further illustrates the specific directionality exhibited by these immune cells in relation to the six aforementioned lymphomas. Surprisingly, all immune cells exhibit consistent directional activity across the six different lymphomas, including CD28^−^CD25^++^CD8br%CD8br, which is identified as a risk factor for the four lymphomas.

### 3.3 LDSC Results Causally Associated With Lymphoma

Furthermore, to delve deeper into the correlation between different immune cell populations and lymphoma, we conducted LDSC analysis. Detailed results can be found in the Table S4. Notably, the association of CD28^−^CD25^++^CD8br%CD8br immune cells with all four lymphoma types is particularly noteworthy (*p* = 0.0343 with other and unspecified non‐Hodgkin lymphoma, *p* = 0.0294 with follicular lymphoma, *p* = 0.0306 with non‐follicular lymphoma, *p* = 0.0247 with DLBCL). This finding significantly strengthens the reliability of our previous Mendelian randomisation (MR) results. Moreover, certain immune cell populations, such as CD8br%leukocyte and CD25 on secreting Treg cells, were observed to be associated with three types of lymphoma simultaneously.

### 3.4 Immune Cell SMR Results and LDSC Results Causally Associated With Lymphoma

**FIGURE 4:**
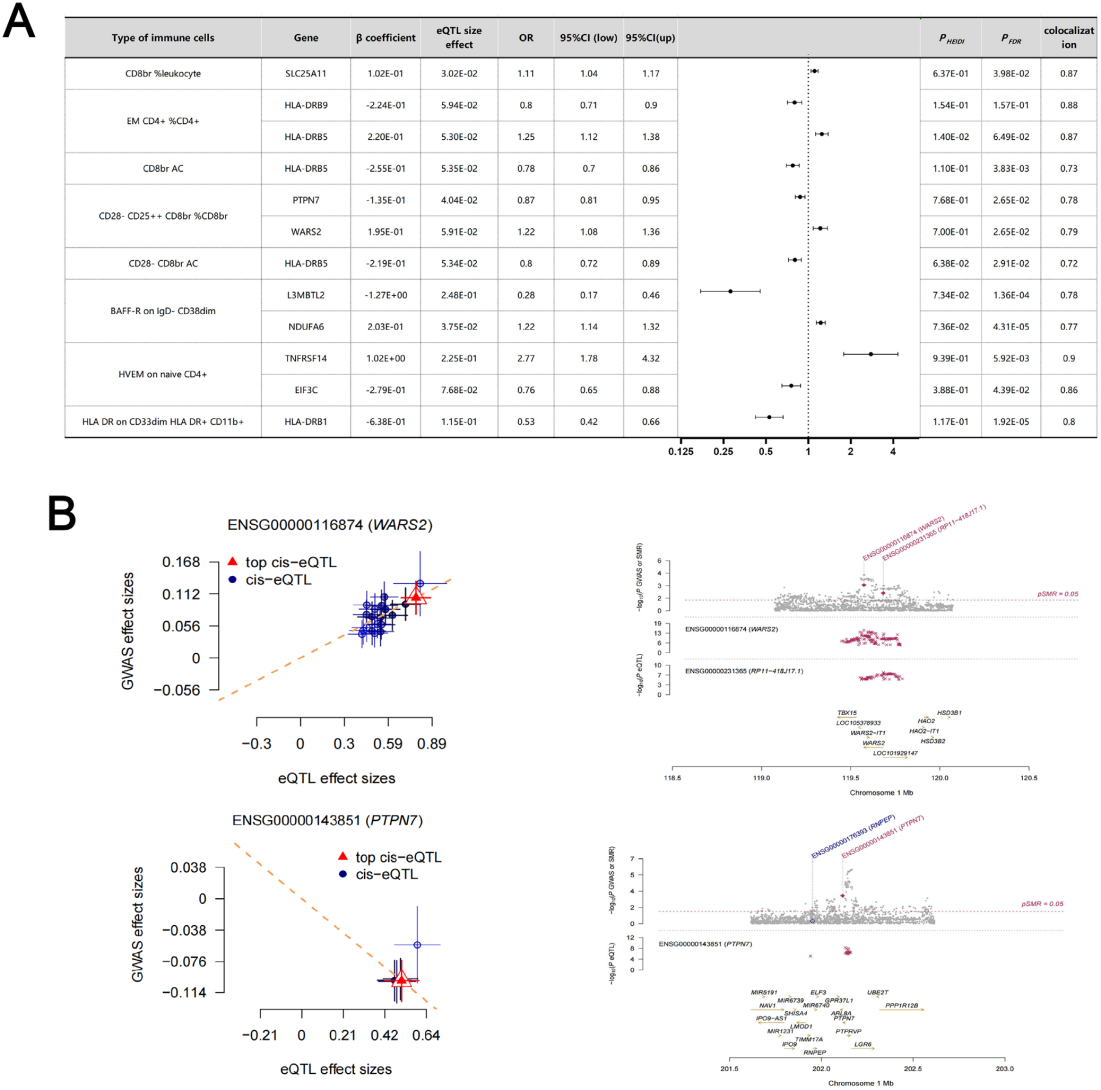
Exploration of potential lymphoma therapeutic genes through SMR analysis. (A) SMR and colocalization results from eQTL of diverse immune cells causally associated with lymphoma. positive correlation is indicated by *β* > 0, while a negative correlation is indicated by *β* < 0. Ratios were computed based on the expected value of the causal estimate (*β* coefficient). Co‐localization was determined by PP.H4 between eQTL and lymphoma, with a PP.H4 threshold of > 0.75 considered as strong evidence for co‐localization. The displayed results are limited to those with PP.H4 values of 0.70 or higher; (B) The associations of PTPN7 and WARS2 with CD28^−^CD25^++^CD8br%CD8br were investigated using eQTL data obtained from SMR analysis of lymphomas.

The associations between immune cells from the lymphoid tissue that were causal for different lymphomas were obtained by SMR analysis (Figure 4A). Through SMR analysis, we identified genes that exhibited a significant association with lymphoma‐associated immune cells (P_FDR_ < 0.05, P_HIED_ > 0.05). Detailed genetic information can be found in Table S5. As shown in Figure 4, we focused on immune cells (CD28^−^CD25^++^ CD8br%CD8br) that were causally associated with all four lymphomas and found WARS2 (beta_SMR[SE]_ = −0.13_[0.04]_, P_SMR_ = 8.37 × 10^−04^, P_FDR_ = 0.026, P_HEID_ = 0.77, Figure 4A), PTPN7 (beta_SMR[SE]_ = 0.19_[0.05]_, P_SMR_ = 9.81 × 10^−04^, P_FDR_ = 0.026, P_HEID_ = 0.69, Figure 4B). Additionally, we have also identified associations between several genes (HLA‐DRB5, TSPAN32, SLC25A11, MRPL28, L3MBTL2, etc.) and diverse immune cell populations, suggesting their potential involvement in the regulation of multiple immune cell types simultaneously.

## 4 Discussion

Using published genetic data, we investigated causal links between immune cell traits and lymphoma subtypes. To the best of our knowledge, this study represents the first Mendelian Randomization analysis that integrates two‐sample MR and SMR approaches to investigate the causal association between multiple immunophenotypes and various lymphomas. Additionally, we employed LDSC analyses to further enhance the robustness and reliability of our findings. The innovative study provides important insights into the interactions between the immune system and lymphomas, offering valuable insights that could inform future prevention and treatment strategies.

Our study unveiled distinct immune cell populations with causal relationships between different subtypes of lymphoma. To further investigate the causal relationship between these immune cells and different lymphomas, we employed a Manhattan plot analysis to examine the genetic architecture of the immune cells associated with these six lymphomas and identify the effector genes linked to them. Our findings revealed a robust association between CD247 and Hodgkin lymphoma, non‐follicular lymphoma, DLBCL, as well as mature T/NK cell lymphomas. Notably, CD247 exhibited predominantly low expression levels in natural killer (NK) and T cells. Interestingly, previous studies conducted in 2015 also reported reduced expression of CD247 in NK/T‐cell lymphoma [19]. The subsequent discovery made by scholars unveiled a significant association between the high expression of CD247 in DLBCL and enhanced overall survival [20]. Our studies consistently support the aforementioned perspective. However, investigations regarding the involvement of CD247 in the other two lymphomas have not been conducted thus far. Consequently, our findings suggest that CD247 may also influence Hodgkin lymphoma and non‐follicular lymphoma. Furthermore, our study revealed a significant correlation between ENTPD1 (ectonucleoside triphosphate diphosphate hydrolase‐1) and Hodgkin lymphoma as well as other unspecified types of non‐Hodgkin lymphoma. The present study demonstrated that inhibiting ENTPD1 can enhance the anticancer efficacy of ceritinib in triple negative breast cancer, melanoma cells, and non‐small cell lung cancer [21]. Our findings demonstrate a significant and robust correlation between ENTPD1 and Hodgkin lymphoma, suggesting that the inhibition of ENTPD1 may also exhibit antitumor effects in this disease. Furthermore, our study identifies CIITA, LYZ, MBL2, and CD40 as potential therapeutic targets for various types of lymphoma treatment [22, 23, 24, 25].

Analysis of immune cells overlapping in causal relationships with lymphoma subtypes revealed seven cell phenotypes significantly associated with three or more lymphoma entities (FDR < 0.05), suggesting their potential as pan‐lymphoma therapeutic targets. Notably, CD28^−^CD25^++^CD8br%CD8br cells emerged as shared risk factors (IVW *β* = 1.12–1.38, *p* < 2.3 × 10^−5^) for four lymphoma subtypes: unspecified non‐Hodgkin lymphoma, DLBCL, follicular lymphoma, and non‐follicular lymphoma. No studies have reported this gene's role in lymphoma. To strengthen the robustness of our findings, we conducted additional LDSC analysis and identified a genetic association between CD28^−^CD25^++^CD8br%CD8br immune cells and the four lymphomas, thereby reinforcing confidence in our initial Mendelian analysis results. Through SMR analysis, we identified WARS2 and PTPN7 as key regulators of CD28^−^CD25^++^CD8br%CD8br immune cells and various lymphomas. PTPN7, a member of the non‐receptor protein tyrosine phosphatase (PTPN) family primarily involved in tyrosine phosphorylation, has been identified as being associated with immune‐related tumours in various cancers, including breast cancer. This discovery positions it as a promising predictive biomarker for immunotherapy [26]. The latest study has unveiled a notable upregulation of WARS2 in hepatocellular carcinoma cells, indicating its potential as an immunotherapeutic target against liver cancer. However, the current literature lacks any reports on the involvement of this gene in lymphoma [27]. Furthermore, by conducting summary‐data‐based Mendelian randomisation (SMR) analysis, we have identified novel genes that regulate immune cell function, including PSPHP1 and AGAP6, which have not been previously investigated in the context of lymphoma. Our findings suggest that these newly discovered gene loci hold promise as innovative therapeutic targets for lymphoma treatment.

This study utilised multi‐modal Mendelian randomisation (MR) approaches to systematically evaluate bidirectional causal relationships between 731 immune cell phenotypes and six lymphoma subtypes using summary statistics from genome‐wide association studies (GWAS) involving 150,000 European‐ancestry individuals. By integrating inverse‐variance weighted (IVW) MR, summary‐data‐based MR (SMR), and linkage disequilibrium score regression (LDSC), this analysis provided robust evidence for immune‐mediated mechanisms in lymphoma pathogenesis while accounting for population stratification and polygenic confounding. These findings contribute to understanding lymphoma aetiology by identifying novel immune regulatory pathways that may inform therapeutic development.

Notwithstanding these advances, several limitations require careful interpretation. First, the extensive scope of analysis (4386 trait‐disease comparisons) necessitated stringent statistical correction via the random‐effects IVW model, which may have reduced sensitivity to small‐to‐moderate effect sizes, particularly in rare lymphoma subtypes. Second, the absence of individual‐level demographic covariates (age, sex, ancestry) restricted subgroup analysis and limited generalisability to European populations. Third, residual pleiotropy cannot be fully excluded despite multiple method triangulation, highlighting the need for functional validation of prioritised immune‐lymphoma pairs. Replication in multi‐ethnic cohorts and mechanistic studies is essential to address these limitations and enhance translational impact.

## 5 Conclusion

In summary, Here, we hypothesise that specific immune cell populations drive lymphoma development in a subtype‐specific manner. Using genetic tools, we aim to: (1) establish causal immune cell‐lymphoma relationships; (2) identify effector genes; and (3) validate genetic correlations. Through SMR analysis, we confirmed associated gene loci that are causally linked to different lymphomas, enabling us to provide a detailed risk profile for each subtype. These findings provide novel genetic evidence for immune cell‐mediated lymphoma pathogenesis and highlight potential therapeutic targets.

## Author Contributions

**Jingxuan Lian:** formal analysis (equal), methodology (equal), software (equal), writing – original draft (lead). **Xinghong Zhang:** project administration (equal), resources (equal), software (equal), visualization (equal). **Wenjie Chen:** formal analysis (equal), investigation (equal), resources (equal). **Zheshen Lin:** data curation (equal), investigation (equal). **Ming Lu:** software (equal), supervision (equal), validation (equal). **Rong Liang:** conceptualization (lead), writing – review and editing (lead).

## Ethics Statement

Ethical approval was not needed since all data used had been previously published in the public database.

## Consent

The authors have nothing to report.

## Conflicts of Interest

The authors declare no conflicts of interest.

## Supporting information


Table S1.

## Data Availability

All data used in the current study are publicly available GWAS summary data (https://gwas.mrcieu.ac.uk/).
