# Supplementary material for: Revealing Putative Causal Genes by Establishing the Causality Between Different Lymphomas and Immune Cells
Source: J Cell Mol Med. 2025 May 13;29(9):e70535. doi: 10.1111/jcmm.70535 (PMC12074867; doi:10.1111/jcmm.70535)
Supplement: Supplementary file 1 — Table S1. [file JCMM-29-e70535-s001.docx]

| **Supplementary material Table 1. Causal relationship between immune cells and different lymphomas** | | | | | | | | | | |
| --- | --- | --- | --- | --- | --- | --- | --- | --- | --- | --- |
| **outcome** | **id.exposure** | **exposure** | **panel** | **nsnp** | **pval** | **OR** | **95%CI(low)** | **95%CI(up)** | **p_FDR** |  |
| **Hodgkin lymphoma** | GCST90001625 | HLA DR+ CD4+ %T cell | TBNK | 12 | 7.45E-05 | 0.539 | 0.399 | 0.679 | 0.027 |  |
|  | GCST90001874 | HVEM on TD CD8br | Maturation stages of T cell | 14 | 3.96E-04 | 0.788 | 0.731 | 0.845 | 0.097 |  |
|  | GCST90001804 | CD27 on IgD- CD38dim | B cell | 39 | 7.06E-04 | 1.226 | 1.175 | 1.278 | 0.074 |  |
|  | GCST90001838 | CD3 on naive CD8br | Maturation stages of T cell | 32 | 7.26E-04 | 1.193 | 1.148 | 1.237 | 0.066 |  |
|  | GCST90001457 | CD11c+ HLA DR++ monocyte %monocyte | cDC | 10 | 9.58E-04 | 0.629 | 0.496 | 0.762 | 0.078 |  |
|  | GCST90001844 | CD3 on TD CD4+ | Maturation stages of T cell | 13 | 1.35E-03 | 1.288 | 1.214 | 1.362 | 0.09 |  |
|  | GCST90001941 | CD25 on secreting Treg | Treg | 11 | 1.48E-03 | 1.223 | 1.162 | 1.284 | 0.09 |  |
|  | GCST90001641 | CD3- lymphocyte %leukocyte | TBNK | 8 | 1.54E-03 | 1.58 | 1.451 | 1.709 | 0.087 |  |
|  | GCST90001895 | CD28 on CD28+ DN (CD4-CD8-) | Treg | 2 | 1.69E-03 | 0.645 | 0.521 | 0.769 | 0.082 |  |
|  | GCST90001989 | CD40 on CD14- CD16+ monocyte | Monocyte | 39 | 1.70E-03 | 0.863 | 0.823 | 0.903 | 0.078 |  |
|  | GCST90001626 | HLA DR+ CD4+ %lymphocyte | TBNK | 15 | 1.92E-03 | 0.667 | 0.556 | 0.778 | 0.083 |  |
| **Diffuse large B-cell lymphoma** | GCST90002113 | HLA DR on HLA DR+ T cell | TBNK | 6 | 3.20E-05 | 0.514 | 0.343 | 0.685 | 0.023 |  |
|  | GCST90001869 | CD3 on resting Treg | Treg | 44 | 3.30E-05 | 0.81 | 0.756 | 0.863 | 0.012 |  |
|  | GCST90002047 | CD45 on Gr MDSC | Myeloid cell | 8 | 4.31E-05 | 0.654 | 0.555 | 0.752 | 0.011 |  |
|  | GCST90001677 | CD28- CD25++ CD8br %CD8br | Treg | 14 | 9.50E-05 | 1.681 | 1.552 | 1.809 | 0.017 |  |
|  | GCST90001413 | IgD- CD24- %B cell | B cell | 12 | 1.52E-04 | 0.779 | 0.707 | 0.852 | 0.022 |  |
|  | GCST90001687 | CD28- CD8br AC | Treg | 15 | 1.64E-04 | 1.947 | 1.762 | 2.132 | 0.02 |  |
|  | GCST90001865 | CD3 on CD28- CD8br | Treg | 11 | 1.78E-04 | 1.7 | 1.573 | 1.827 | 0.019 |  |
|  | GCST90001989 | CD40 on CD14- CD16+ monocyte | Monocyte | 39 | 2.48E-04 | 0.844 | 0.796 | 0.893 | 0.023 |  |
|  | GCST90001870 | CD34 on HSC | Myeloid cell | 13 | 3.29E-04 | 0.792 | 0.725 | 0.86 | 0.027 |  |
|  | GCST90001877 | HVEM on naive CD4+ | Maturation stages of T cell | 9 | 4.02E-04 | 0.72 | 0.625 | 0.816 | 0.029 |  |
|  | GCST90001941 | CD25 on secreting Treg | Treg | 11 | 5.01E-04 | 1.312 | 1.233 | 1.391 | 0.033 |  |
|  | GCST90002023 | CD4 on CM CD4 + | Maturation stages of T cell | 10 | 5.83E-04 | 0.636 | 0.508 | 0.764 | 0.036 |  |
|  | GCST90002073 | SSC-A on monocyte | cDC | 32 | 6.39E-04 | 1.279 | 1.213 | 1.346 | 0.036 |  |
|  | GCST90001613 | DN (CD4-CD8-) %leukocyte | TBNK | 14 | 1.26E-03 | 0.803 | 0.743 | 0.862 | 0.066 |  |
|  | GCST90001928 | CD127 on CD28+ CD45RA- CD8br | Treg | 9 | 1.43E-03 | 0.708 | 0.605 | 0.81 | 0.07 |  |
|  | GCST90002110 | HLA DR on CD33dim HLA DR+ CD11b+ | Myeloid cell | 24 | 1.44E-03 | 0.848 | 0.801 | 0.894 | 0.066 |  |
|  | GCST90001640 | CD3- lymphocyte %lymphocyte | TBNK | 3 | 1.83E-03 | 0.237 | 0.136 | 0.61 | 0.079 |  |
|  | GCST90001596 | CD8dim AC | TBNK | 13 | 1.92E-03 | 0.734 | 0.649 | 0.819 | 0.078 |  |
|  | GCST90002074 | SSC-A on CD14+ monocyte | TBNK | 32 | 2.84E-03 | 1.286 | 1.212 | 1.36 | 0.109 |  |
| **other and unspecified types of non-Hodgkin lymphoma** | GCST90001714 | BAFF-R on IgD- CD38dim | B cell | 2 | 5.21E-05 | 0.522 | 0.722 | 0.738 | 0.038 |  |
|  | GCST90001543 | EM CD4+ %CD4+ | Maturation stages of T cell | 19 | 6.85E-05 | 0.744 | 0.843 | 0.852 | 0.025 |  |
|  | GCST90001678 | CD28- CD25++ CD8br AC | Treg | 14 | 1.07E-04 | 1.267 | 1.392 | 1.404 | 0.026 |  |
|  | GCST90001826 | IgD on unsw mem | B cell | 27 | 2.62E-04 | 0.754 | 0.856 | 0.866 | 0.048 |  |
|  | GCST90001687 | CD28- CD8br AC | Treg | 15 | 3.28E-04 | 1.378 | 1.56 | 1.58 | 0.048 |  |
|  | GCST90001682 | CD127- CD8br %T cell | Treg | 6 | 3.92E-04 | 1.473 | 1.722 | 1.759 | 0.041 |  |
|  | GCST90001420 | IgD- CD38br AC | B cell | 11 | 4.14E-04 | 0.804 | 0.892 | 0.905 | 0.038 |  |
|  | GCST90001630 | CD8br NKT AC | TBNK | 18 | 4.20E-04 | 1.331 | 1.499 | 1.518 | 0.034 |  |
|  | GCST90001607 | CD8br %leukocyte | TBNK | 13 | 7.14E-04 | 1.287 | 1.465 | 1.496 | 0.052 |  |
|  | GCST90001679 | CD25++ CD8br %T cell | Treg | 10 | 8.43E-04 | 0.701 | 0.847 | 0.874 | 0.056 |  |
|  | GCST90001729 | CD19 on IgD+ CD38br | B cell | 15 | 8.82E-04 | 1.022 | 1.038 | 1.04 | 0.054 |  |
|  | GCST90001625 | HLA DR+ CD4+ %T cell | TBNK | 12 | 9.54E-04 | 0.63 | 0.816 | 0.85 | 0.054 |  |
|  | GCST90001827 | IgD on IgD+ | B cell | 19 | 9.57E-04 | 0.709 | 0.855 | 0.885 | 0.05 |  |
|  | GCST90002074 | SSC-A on CD14+ monocyte | TBNK | 32 | 9.58E-04 | 1.116 | 1.205 | 1.224 | 0.047 |  |
|  | GCST90001870 | CD34 on HSC | Myeloid cell | 13 | 9.62E-04 | 0.81 | 0.897 | 0.912 | 0.044 |  |
|  | GCST90001677 | CD28- CD25++ CD8br %CD8br | Treg | 14 | 1.04E-03 | 1.246 | 1.415 | 1.449 | 0.045 |  |
|  | GCST90001877 | HVEM on naive CD4+ | Maturation stages of T cell | 9 | 1.55E-03 | 0.776 | 0.885 | 0.906 | 0.063 |  |
|  | GCST90001399 | IgD- CD27- %B cell | B cell | 15 | 2.51E-03 | 0.701 | 0.849 | 0.878 | 0.096 |  |
|  | GCST90001774 | CD24 on transitional | B cell | 12 | 2.60E-03 | 0.67 | 0.831 | 0.859 | 0.095 |  |
| **mature T/NK-cell lymphomas** | GCST90001838 | CD3 on naive CD8br | Maturation stages of T cell | 32 | 1.60E-04 | 0.797 | 0.734 | 0.86 | 0.059 |  |
|  | GCST90001846 | CD3 on CM CD8br | Maturation stages of T cell | 22 | 2.20E-04 | 0.674 | 0.57 | 0.778 | 0.054 |  |
|  | GCST90001885 | CD16-CD56 on HLA DR+ NK | TBNK | 15 | 2.27E-04 | 0.695 | 0.607 | 0.783 | 0.041 |  |
|  | GCST90001414 | IgD- CD24- AC | B cell | 10 | 3.06E-04 | 0.614 | 0.478 | 0.75 | 0.045 |  |
|  | GCST90001900 | CD28 on resting Treg | Treg | 10 | 4.97E-04 | 0.626 | 0.495 | 0.758 | 0.045 |  |
|  | GCST90001457 | CD11c+ HLA DR++ monocyte %monocyte | cDC | 10 | 6.54E-04 | 0.545 | 0.368 | 0.723 | 0.048 |  |
|  | GCST90001865 | CD3 on CD28- CD8br | Treg | 11 | 6.66E-04 | 0.607 | 0.461 | 0.753 | 0.044 |  |
|  | GCST90001830 | BAFF-R on CD20- | B cell | 13 | 7.62E-04 | 1.471 | 1.361 | 1.581 | 0.046 |  |
|  | GCST90001774 | CD24 on transitional | B cell | 12 | 7.62E-04 | 0.591 | 0.441 | 0.74 | 0.043 |  |
|  | GCST90001413 | IgD- CD24- %B cell | B cell | 12 | 7.72E-04 | 0.756 | 0.672 | 0.841 | 0.04 |  |
|  | GCST90001420 | IgD- CD38br AC | B cell | 11 | 8.51E-04 | 0.745 | 0.665 | 0.826 | 0.041 |  |
|  | GCST90001868 | CD3 on CD4 Treg | Treg | 28 | 8.68E-04 | 0.794 | 0.724 | 0.864 | 0.04 |  |
|  | GCST90001864 | CD3 on CD28+ CD45RA+ CD8br | Treg | 37 | 9.02E-04 | 0.81 | 0.748 | 0.872 | 0.039 |  |
|  | GCST90001734 | CD19 on IgD- CD38br | B cell | 10 | 1.05E-03 | 0.611 | 0.463 | 0.759 | 0.04 |  |
| **non-follicular lymphoma** | GCST90001826 | IgD on unsw mem | B cell | 27 | 6.29E-05 | 0.853 | 0.819 | 0.887 | 0.046 |  |
|  | GCST90001900 | CD28 on resting Treg | Treg | 10 | 1.56E-04 | 0.86 | 0.815 | 0.905 | 0.057 |  |
|  | GCST90002110 | HLA DR on CD33dim HLA DR+ CD11b+ | Myeloid cell | 24 | 1.67E-04 | 0.899 | 0.875 | 0.923 | 0.041 |  |
|  | GCST90002062 | CD4 on CD28+ CD4+ | Treg | 11 | 2.22E-04 | 0.774 | 0.715 | 0.833 | 0.041 |  |
|  | GCST90001630 | CD8br NKT AC | TBNK | 18 | 4.74E-04 | 1.171 | 1.124 | 1.219 | 0.069 |  |
|  | GCST90001827 | IgD on IgD+ | B cell | 19 | 5.37E-04 | 0.875 | 0.842 | 0.908 | 0.065 |  |
|  | GCST90001677 | CD28- CD25++ CD8br %CD8br | Treg | 14 | 6.43E-04 | 1.17 | 1.123 | 1.218 | 0.067 |  |
|  | GCST90002112 | HLA DR on CD33- HLA DR+ | Myeloid cell | 20 | 6.91E-04 | 0.928 | 0.905 | 0.95 | 0.063 |  |
|  | GCST90001870 | CD34 on HSC | Myeloid cell | 13 | 7.22E-04 | 0.91 | 0.883 | 0.938 | 0.059 |  |
|  | GCST90001607 | CD8br %leukocyte | TBNK | 13 | 7.42E-04 | 1.299 | 1.231 | 1.367 | 0.054 |  |
|  | GCST90001592 | CD8br AC | TBNK | 22 | 9.87E-04 | 1.154 | 1.112 | 1.197 | 0.06 |  |
|  | GCST90002074 | SSC-A on CD14+ monocyte | TBNK | 32 | 1.33E-03 | 1.118 | 1.088 | 1.147 | 0.075 |  |
|  | GCST90001729 | CD19 on IgD+ CD38br | B cell | 15 | 1.65E-03 | 1.017 | 1.012 | 1.022 | 0.086 |  |
|  | GCST90002106 | HLA DR on DC | cDC | 37 | 1.77E-03 | 0.925 | 0.904 | 0.946 | 0.086 |  |
|  | GCST90001867 | CD3 on CD4+ | Treg | 29 | 1.88E-03 | 0.88 | 0.845 | 0.915 | 0.086 |  |
|  | GCST90001457 | CD11c+ HLA DR++ monocyte %monocyte | cDC | 10 | 2.09E-03 | 0.805 | 0.743 | 0.867 | 0.09 |  |
|  | GCST90001824 | IgD on IgD+ CD38br | B cell | 29 | 2.71E-03 | 0.905 | 0.877 | 0.934 | 0.099 |  |
|  | GCST90001941 | CD25 on secreting Treg | Treg | 11 | 2.83E-03 | 1.121 | 1.089 | 1.154 | 0.098 |  |
| **follicular lymphoma** | GCST90001677 | CD28- CD25++ CD8br %CD8br | Treg | 14 | 2.77E-04 | 1.428 | 1.345 | 1.512 | 0.067 |  |
|  | GCST90001844 | CD3 on TD CD4+ | Maturation stages of T cell | 13 | 2.79E-04 | 1.257 | 1.199 | 1.315 | 0.051 |  |
|  | GCST90002056 | CD8 on EM CD8br | Maturation stages of T cell | 20 | 6.54E-04 | 0.808 | 0.754 | 0.863 | 0.096 |  |
|  | GCST90001988 | HLA DR on CD14+ CD16- monocyte | Monocyte | 26 | 7.74E-04 | 1.186 | 1.143 | 1.23 | 0.094 |  |
|  | GCST90001682 | CD127- CD8br %T cell | Treg | 6 | 1.06E-03 | 1.654 | 1.505 | 1.804 | 0.097 |  |
|  | GCST90001749 | CD20 on IgD+ CD38- naive | B cell | 12 | 1.15E-03 | 1.356 | 1.276 | 1.435 | 0.094 |  |
|  | GCST90002073 | SSC-A on monocyte | cDC | 32 | 1.31E-03 | 1.146 | 1.11 | 1.182 | 0.096 |  |
|  | GCST90001607 | CD8br %leukocyte | TBNK | 13 | 1.32E-03 | 1.429 | 1.334 | 1.523 | 0.088 |  |
|  | GCST90001678 | CD28- CD25++ CD8br AC | Treg | 14 | 1.44E-03 | 1.251 | 1.191 | 1.311 | 0.088 |  |
|  | GCST90001734 | CD19 on IgD- CD38br | B cell | 10 | 1.73E-03 | 0.729 | 0.643 | 0.815 | 0.091 |  |
|  | GCST90002038 | CD80 on CD62L+ plasmacytoid DC | cDC | 12 | 1.79E-03 | 1.168 | 1.126 | 1.211 | 0.087 |  |
|  | GCST90001839 | CD3 on EM CD8br | Maturation stages of T cell | 20 | 1.97E-03 | 1.216 | 1.161 | 1.272 | 0.09 |  |
|  | GCST90001872 | HVEM on naive CD8br | Maturation stages of T cell | 12 | 2.07E-03 | 0.815 | 0.758 | 0.871 | 0.089 |  |
|  | GCST90001640 | CD3- lymphocyte %lymphocyte | TBNK | 3 | 2.36E-03 | 0.485 | 0.268 | 0.701 | 0.096 |  |
|  | GCST90001895 | CD28 on CD28+ DN (CD4-CD8-) | Treg | 2 | 2.79E-03 | 0.341 | 0.017 | 0.666 | 0.102 |  |
| The MR analysis method employed in the table was inverse variance weighted, and random or fixed effect models were selected based on the magnitude of heterogeneity. Only results with adjusted P<0.10 were presented. In cases where certain outcomes did not meet the criteria (adjusted P<0.10), only the result with the smallest adjusted P value was displayed. OR, odd ratio; CI, condifence interval; | | | | | | | | | | |

| **Supplementary material Table 2. The results of a sensitivity analysis using mendelian randomization were conducted on immune cells.** | | | | | | | | | | | | |
| --- | --- | --- | --- | --- | --- | --- | --- | --- | --- | --- | --- | --- |
| **Disease** | **exposure** | **MR Egger** | | **Inverse variance weighted** | | **I^2** | **F statistic** | **MR.PRESSO** | | **pleiotropy** | | |
|  |  | **Q** | **Pvalue** | **Q** | **Pvalue** |  |  | **Global.Test.** | **Pvalue** | **egger intercept** | **se** | **pval** |
| **Hodgkin lymphoma** | HLA DR+ CD4+ %T cell | 17.94796734 | 0.805525167 | 20.75719127 | 0.706067511 | 0.00% | 10.596 | 21.44578 | 0.519 | 0.078 | 0.116 | 0.503 |
|  | HVEM on TD CD8br | 12.37087845 | 0.135407435 | 17.75808372 | 0.038086218 | 20.30% | 11.319 | 12.11981 | 0.935 | 0.054 | 0.167 | 0.591 |
|  | CD27 on IgD- CD38dim | 27.37995592 | 0.006810066 | 28.12827176 | 0.008684214 | 48.60% | 17.522 | 11.52621 | 0.929 | 0.059 | 0.196 | 0.529 |
|  | CD3 on naive CD8br | 26.62623216 | 0.03193342 | 28.70293662 | 0.026017875 | 61.20% | 33.665 | 23.76851 | 0.0213 | 0.07 | 0.141 | 0.941 |
|  | CD11c+ HLA DR++ monocyte %monocyte | 10.6734708 | 0.013629158 | 12.46589627 | 0.014203009 | 39.80% | 38.21 | 41.26703 | 0.229 | 0.078 | 0.156 | 0.562 |
|  | CD3 on TD CD4+ | 22.28018101 | 0.134373088 | 23.69328461 | 0.128011158 | 10.20% | 21.155 | 34.05047 | 0.091 | -0.103 | 0.086 | 0.27 |
|  | CD25 on secreting Treg | 9.168866856 | 0.606308465 | 6.963134849 | 0.728920607 | 0.00% | 4.799 | 21.09836 | 0.688 | 0.066 | 0.096 | 0.191 |
|  | CD3- lymphocyte %leukocyte | 13.46604499 | 0.096788898 | 13.65459403 | 0.13515337 | 22.10% | 12.835 | 18.70186 | 0.112 | -0.008 | 0.15 | 0.632 |
|  | CD28 on CD28+ DN (CD4-CD8-) | 7.686529203 | 0.566018823 | 6.133508577 | 0.909202808 | 0.00% | 4.799 | 31.56814 | 0.444 | NA | NA | NA |
|  | CD40 on CD14- CD16+ monocyte | 31.2077251 | 0.018840983 | 32.34101998 | 0.020028264 | 45.70% | 75.583 | 11.96788 | 0.944 | -0.023 | 0.092 | 0.791 |
|  | HLA DR+ CD4+ %lymphocyte | 33.64843272 | 0.039503487 | 34.3679526 | 0.045049971 | 34.20% | 25.829 | 19.76787 | 0.561 | -0.139 | 0.108 | 0.69 |
| **Diffuse large B-cell lymphoma** | HLA DR on HLA DR+ T cell | 12.4124671 | 0.413147822 | 18.0610753 | 0.080174227 | 11.90% | 23.479 | 23.47166 | 0.525 | 0.008 | 0.054 | 0.928 |
|  | CD3 on resting Treg | 6.33758789 | 0.78614811 | 7.8834828 | 0.723701725 | 0.00% | 10.458 | 9.71083 | 0.7883 | 0.032 | 0.046 | 0.733 |
|  | CD45 on Gr MDSC | 14.89080249 | 0.314219281 | 15.08050608 | 0.37267362 | 13.30% | 16.947 | 15.3753 | 0.7386 | -0.191 | 0.156 | 0.834 |
|  | CD28- CD25++ CD8br %CD8br | 4.653994647 | 0.946759447 | 5.114614819 | 0.954040685 | 0.00% | 11.139 | 20.73561 | 0.963 | 0.029 | 0.13 | 0.913 |
|  | IgD- CD24- %B cell | 2.946038147 | 0.982665566 | 5.109136467 | 0.925766088 | 0.00% | 24.487 | 27.8365 | 0.789 | -0.02 | 0.059 | 0.172 |
|  | CD28- CD8br AC | 10.63408745 | 0.560510003 | 9.177986319 | 0.240128768 | 17.00% | 18.074 | 19.36655 | 0.5573 | 0.08 | 0.048 | 0.836 |
|  | CD3 on CD28- CD8br | 21.79824774 | 0.039844173 | 21.8260487 | 0.058116328 | 56.50% | 34.445 | 23.23645 | 0.3757 | -0.039 | 0.03 | 0.464 |
|  | CD40 on CD14- CD16+ monocyte | 20.69381892 | 0.079209462 | 20.69727621 | 0.109645549 | 25.80% | 13.472 | 27.06498 | 0.265 | -0.06 | 0.022 | 0.17 |
|  | CD34 on HSC | 25.96136612 | 0.100644559 | 29.80894291 | 0.054280868 | 24.70% | 19.919 | 9.26457 | 0.187 | -0.065 | 0.047 | 0.341 |
|  | HVEM on naive CD4+ | 39.72790472 | 0.011630483 | 39.91469355 | 0.015710869 | 57.50% | 30.587 | 16.26457 | 0.0946 | 0.039 | 0.038 | 0.939 |
|  | CD25 on secreting Treg | 23.80268084 | 0.013591229 | 24.87589195 | 0.015422057 | 53.80% | 23.788 | 19.23465 | 0.1836 | -0.05 | 0.03 | 0.498 |
|  | CD4 on CM CD4 + | 11.13273644 | 0.025112054 | 11.37318632 | 0.04446278 | 38.80% | 21.247 | 18.26457 | 0.287 | 0.01 | 0.033 | 0.957 |
|  | SSC-A on monocyte | 22.32401241 | 0.02197986 | 23.06051629 | 0.027216549 | 55.70% | 86.356 | 2.61545 | 0.089 | 0.02 | 0.036 | 0.361 |
|  | DN (CD4-CD8-) %leukocyte | 25.26623577 | 0.032028232 | 25.59881365 | 0.04245705 | 49.50% | 23.763 | 62.13265 | 0.186 | -0.027 | 0.029 | 0.99 |
|  | CD127 on CD28+ CD45RA- CD8br | 11.56924304 | 0.238696426 | 13.38555084 | 0.202906782 | 18.40% | 49.595 | 54.23165 | 0.325 | -0.056 | 0.035 | 0.238 |
|  | HLA DR on CD33dim HLA DR+ CD11b+ | 14.50010463 | 0.269918144 | 14.50048342 | 0.339564592 | 12.60% | 27.956 | 15.23675 | 0.642 | -0.01 | 0.025 | 0.6 |
|  | CD3- lymphocyte %lymphocyte | 5.338437417 | 0.913675285 | 6.051991912 | 0.913438499 | 0.00% | 41.277 | 32.31249 | 0.968 | -0.088 | 0.062 | 0.659 |
|  | CD8dim AC | 23.46478659 | 0.053114722 | 23.48305037 | 0.074408455 | 33.60% | 16.564 | 30.61535 | 0.446 | -0.023 | 0.048 | 0.304 |
|  | SSC-A on CD14+ monocyte | 24.04279103 | 0.088574051 | 25.19731816 | 0.090379365 | 33.10% | 25.394 | 19.13575 | 0.346 | 0.035 | 0.135 | 0.941 |
| **other and unspecified types of non-Hodgkin lymphoma** | BAFF-R on IgD- CD38dim | 4.384243385 | 0.884353893 | 2.829485417 | 0.944598118 | 0.0%. | 8.564 | 20.76519 | 0.499 | NA | NA | NA |
|  | EM CD4+ %CD4+ | 30.12334032 | 0.05024957 | 34.52074861 | 0.022810424 | 42.20% | 19.75 | 18.32651 | 0.229 | 0.053 | 0.105 | 0.725 |
|  | CD28- CD25++ CD8br AC | 33.12814627 | 0.101398354 | 34.01795245 | 0.10752468 | 7.30% | 16.181 | 18.23499 | 0.768 | 0.051 | 0.085 | 0.805 |
|  | IgD on unsw mem | 6.009953996 | 0.872699267 | 6.477149093 | 0.890147682 | 0.00%. | 7.149 | 9.03266 | 0.445 | -0.012 | 0.022 | 0.236 |
|  | CD28- CD8br AC | 11.02970172 | 0.526374972 | 11.03598072 | 0.607803287 | 0.00% | 25.783 | 13.61523 | 0.666 | -0.018 | 0.013 | 0.736 |
|  | CD127- CD8br %T cell | 7.754189101 | 0.55909535 | 7.824282631 | 0.645996026 | 0.00% | 9.679 | 25.23166 | 0.519 | 0.012 | 0.023 | 0.378 |
|  | IgD- CD38br AC | 30.24482402 | 0.006677339 | 30.29348743 | 0.005430453 | 62.8%. | 32.233 | 27.24613 | 0.664 | 0.005 | 0.015 | 0.796 |
|  | CD8br NKT AC | 19.63166483 | 0.237270266 | 20.04933224 | 0.093986975 | 10.10% | 55.1 | 30.13296 | 0.665 | 0.001 | 0.025 | 0.568 |
|  | CD8br %leukocyte | 5.933314612 | 0.877776408 | 6.382812469 | 0.895569037 | 0.00% | 19.346 | 38.23265 | 0.329 | -0.018 | 0.016 | 0.933 |
|  | CD25++ CD8br %T cell | 10.49483827 | 0.032868166 | 10.51027843 | 0.06200234 | 29.30% | 28.457 | 15.23295 | 0.958 | -0.055 | 0.033 | 0.306 |
|  | CD19 on IgD+ CD38br | 6.779676954 | 0.871823313 | 6.869093096 | 0.908770998 | 0.00% | 13.206 | 9.2317 | 0.788 | 0.017 | 0.023 | 0.899 |
|  | HLA DR+ CD4+ %T cell | 17.78890847 | 0.037703075 | 17.78983965 | 0.05861393 | 48.60% | 17.522 | 26.23165 | 0.864 | -0.035 | 0.031 | 0.818 |
|  | IgD on IgD+ | 23.36080233 | 0.05464627 | 27.14533476 | 0.027574284 | 30.70% | 27.784 | 26.26945 | 0.113 | -0.004 | 0.02 | 0.649 |
|  | SSC-A on CD14+ monocyte | 23.80268084 | 0.013591229 | 24.87589195 | 0.015422057 | 59.70% | 97.654 | 40.85165 | 0.236 | 0.008 | 0.04 | 0.397 |
|  | CD34 on HSC | 22.94026513 | 0.115340716 | 23.03845807 | 0.148002851 | 10.20% | 21.155 | 38.23265 | 0.469 | 0.01 | 0.021 | 0.967 |
|  | CD28- CD25++ CD8br %CD8br | 44.21472004 | 0.001408397 | 44.21472025 | 0.002193011 | 61.20% | 39.67 | 15.26266 | 0.095 | 0.009 | 0.013 | 0.134 |
|  | HVEM on naive CD4+ | 30.27544794 | 0.086591085 | 30.275452 | 0.111921705 | 22.10% | 12.835 | 11.38563 | 0.139 | 0.012 | 0.008 | 0.415 |
|  | IgD- CD27- %B cell | 25.75354009 | 0.079069351 | 25.82475306 | 0.103832942 | 19.00% | 10.24 | 19.84612 | 0.239 | 0.006 | 0.02 | 0.554 |
|  | CD24 on transitional | 30.07456658 | 0.050857106 | 30.08750019 | 0.068448196 | 14.70% | 9.574 | 16.61948 | 0.554 | 0.008 | 0.015 | 0.637 |
| **mature T/NK-cell lymphomas** | CD3 on naive CD8br | 21.06378746 | 0.020653136 | 22.10700379 | 0.023557252 | 64.20% | 74.583 | 26.26496 | 0.023 | 0.067 | 0.061 | 0.496 |
|  | CD3 on CM CD8br | 23.63375368 | 0.071579756 | 23.91078774 | 0.091471178 | 34.20% | 25.829 | 30.23265 | 0.136 | 0.028 | 0.05 | 0.717 |
|  | CD16-CD56 on HLA DR+ NK | 12.96954154 | 0.966619743 | 13.33234948 | 0.972161906 | 0.00% | 10.298 | 59.77465 | 0.888 | 0.087 | 0.053 | 0.202 |
|  | IgD- CD24- AC | 48.42399184 | 0.051788427 | 48.83187282 | 0.060276368 | 19.80% | 17.461 | 46.13266 | 0.236 | 0.063 | 0.044 | 0.553 |
|  | CD28 on resting Treg | 34.96547425 | 0.422008143 | 35.04972688 | 0.465844563 | 0.00% | 15.241 | 15.78963 | 0.854 | -0.03 | 0.03 | 0.516 |
|  | CD11c+ HLA DR++ monocyte %monocyte | 30.1161361 | 0.050338902 | 30.14201113 | 0.067585018 | 20.30% | 16.315 | 18.56463 | 0.654 | -0.023 | 0.025 | 0.625 |
|  | CD3 on CD28- CD8br | 9.711558024 | 0.285857196 | 9.516516013 | 0.574327485 | 0.00% | 12.404 | 16.16355 | 0.756 | 0.056 | 0.06 | 0.152 |
|  | BAFF-R on CD20- | 7.038456088 | 0.532489291 | 7.08428653 | 0.62834425 | 0.00% | 6.412 | 18.65813 | 0.846 | 0.03 | 0.05 | 0.254 |
|  | CD24 on transitional | 23.54833044 | 0.487652995 | 23.71719289 | 0.535758732 | 0.00% | 10.79 | 15.23265 | 0.456 | -0.034 | 0.047 | 0.831 |
|  | IgD- CD24- %B cell | 29.79401708 | 0.123557428 | 22.94026513 | 0.115340716 | 9.50% | 24.753 | 50.64951 | 0.462 | -0.024 | 0.034 | 0.242 |
|  | IgD- CD38br AC | 18.01625599 | 0.081204183 | 19.04777586 | 0.087381984 | 11.00% | 29.417 | 18764923 | 0.139 | 0.054 | 0.081 | 0.765 |
|  | CD3 on CD4 Treg | 22.74994863 | 0.006783 | 24.03000409 | 0.007521148 | 75.70% | 86.356 | 20.61243 | 0.019 | 0.046 | 0.106 | 0.529 |
|  | CD3 on CD28+ CD45RA+ CD8br | 18.17288673 | 0.005814359 | 19.8350864 | 0.005937127 | 65.70% | 73.715 | 9.23165 | 0.165 | -0.034 | 0.039 | 0.866 |
|  | CD19 on IgD- CD38br | 24.30212779 | 0.042115359 | 24.89052473 | 0.051433124 | 15.40% | 41.7 | 11.6824 | 0.136 | -0.023 | 0.036 | 0.894 |
| **non-follicular lymphoma** | IgD on unsw mem | 38.68149793 | 0.010710979 | 39.90988526 | 0.011076758 | 53.80% | 23.788 | 18.59785 | 0.264 | 0.03 | 0.066 | 0.568 |
|  | CD28 on resting Treg | 17.54849915 | 0.130103125 | 19.99760877 | 0.095269897 | 38.80% | 21.247 | 21.45866 | 0.319 | -0.126 | 0.084 | 0.454 |
|  | HLA DR on CD33dim HLA DR+ CD11b+ | 73.38786501 | 0.002642779 | 73.43535991 | 0.003527773 | 75.70% | 86.356 | 16.56873 | 0.003 | -0.001 | 0.054 | 0.065 |
|  | CD4 on CD28+ CD4+ | 30.94574796 | 0.707615537 | 39.91064487 | 0.342013945 | 0.00% | 12.887 | 7.48856 | 0.964 | -0.043 | 0.077 | 0.308 |
|  | CD8br NKT AC | 20.76367897 | 0.595516816 | 20.76849762 | 0.652341755 | 0.00% | 14.188 | 11.74654 | 0.645 | 0.056 | 0.063 | 0.659 |
|  | IgD on IgD+ | 21.36824459 | 0.558610201 | 25.87737019 | 0.359416532 | 0.00% | 13.615 | 30.43498 | 0.551 | -0.106 | 0.074 | 0.356 |
|  | CD28- CD25++ CD8br %CD8br | 18.26498857 | 0.019325241 | 22.15632667 | 0.008396519 | 61.20% | 41.277 | 64.66887 | 0.664 | 0.07 | 0.044 | 0.062 |
|  | HLA DR on CD33- HLA DR+ | 17.58391601 | 0.040319468 | 19.65685753 | 0.032670481 | 33.60% | 16.564 | 26.60375 | 0.123 | 0.021 | 0.06 | 0.121 |
|  | CD34 on HSC | 22.80989413 | 0.088260408 | 24.17653439 | 0.08571907 | 33.10% | 25.394 | 18.2629 | 0.154 | 0.063 | 0.087 | 0.497 |
|  | CD8br %leukocyte | 44.21472004 | 0.001408397 | 44.21472025 | 0.002193011 | 75.40% | 48.702 | 20.54826 | 0.002 | 0.025 | 0.103 | 0.216 |
|  | CD8br AC | 14.16806813 | 0.0774886 | 14.26079938 | 0.113345827 | 39.80% | 38.21 | 29.46592 | 0.648 | -0.086 | 0.056 | 0.231 |
|  | SSC-A on CD14+ monocyte | 23.91813715 | 0.013082782 | 24.74588482 | 0.016074025 | 54.20% | 26.192 | 19.5659 | 0.461 | -0.066 | 0.066 | 0.557 |
|  | CD19 on IgD+ CD38br | 27.16338335 | 0.075981624 | 27.43761516 | 0.094865496 | 25.50% | 14.758 | 15.326 | 0.319 | 0.228 | 0.137 | 0.854 |
|  | HLA DR on DC | 24.07218429 | 0.004491089 | 25.27682978 | 0.004634693 | 74.20% | 67.601 | 19.23264 | 0.009 | -0.187 | 0.117 | 0.038 |
|  | CD3 on CD4+ | 27.02552254 | 0.012341349 | 27.11813437 | 0.018583849 | 69.20% | 60.3 | 80.2361 | 0.367 | 0.08 | 0.088 | 0.412 |
|  | CD11c+ HLA DR++ monocyte %monocyte | 22.5575324 | 0.311038983 | 22.77592024 | 0.356041294 | 10.60% | 46.055 | 51.4855 | 0.167 | -0.009 | 0.103 | 0.567 |
|  | IgD on IgD+ CD38br | 35.00413702 | 0.000124662 | 23.6471282 | 0.008594179 | 76.00% | 41.665 | 30.2346 | 0.023 | -0.042 | 0.126 | 0.665 |
|  | CD25 on secreting Treg | 14.6121343 | 0.688404417 | 14.64286881 | 0.745009626 | 0.00% | 35.742 | 40.23265 | 0.846 | 0.04 | 0.147 | 0.879 |
| **follicular lymphoma** | CD28- CD25++ CD8br %CD8br | 13.8842005 | 0.084834067 | 15.95840562 | 0.067755284 | 32.30% | 16.24 | 20.3265 | 0.554 | 0.009 | 0.017 | 0.701 |
|  | CD3 on TD CD4+ | 10.80344776 | 0.459873833 | 11.15445377 | 0.51573045 | 0.00% | 12.697 | 21.26465 | 0.884 | 0.01 | 0.029 | 0.924 |
|  | CD8 on EM CD8br | 37.47399828 | 0.086554453 | 41.23689042 | 0.051077936 | 13.30% | 10.382 | 15.23164 | 0.645 | 0.00E+00 | 0.018 | 0.561 |
|  | HLA DR on CD14+ CD16- monocyte | 26.64132726 | 0.031798263 | 26.65784043 | 0.045446435 | 43.60% | 59.385 | 11.27493 | 0.513 | -0.031 | 0.054 | 0.302 |
|  | CD127- CD8br %T cell | 16.39828151 | 0.69162848 | 16.57535087 | 0.736503976 | 0.00% | 6.343 | 18.2326 | 0.319 | -0.003 | 0.037 | 0.52 |
|  | CD20 on IgD+ CD38- naive | 20.96311333 | 0.399306593 | 21.09094142 | 0.453402439 | 0.00% | 19.576 | 9.23294 | 0.629 | 0.055 | 0.043 | 0.382 |
|  | SSC-A on monocyte | 30.86940195 | 0.029804236 | 31.08084438 | 0.039555686 | 62.30% | 70.655 | 26.23165 | 0.136 | -0.004 | 0.023 | 0.231 |
|  | CD8br %leukocyte | 21.66231658 | 0.027123774 | 22.61147824 | 0.031211406 | 48.60% | 17.522 | 27.23656 | 0.394 | -0.001 | 0.039 | 0.606 |
|  | CD28- CD25++ CD8br AC | 7.202527017 | 0.843944531 | 9.296984459 | 0.750161987 | 0.00% | 35.569 | 21.23998 | 0.629 | 0.034 | 0.026 | 0.328 |
|  | CD19 on IgD- CD38br | 8.535068742 | 0.576720072 | 8.574001197 | 0.661148304 | 0.00% | 19.57 | 16.26654 | 0.684 | 0.017 | 0.033 | 0.863 |
|  | CD80 on CD62L+ plasmacytoid DC | 19.96000576 | 0.067846312 | 21.3329285 | 0.066599351 | 10.20% | 21.155 | 29.23325 | 0.254 | -0.004 | 0.023 | 0.233 |
|  | CD3 on EM CD8br | 19.73794743 | 0.031830994 | 20.78872563 | 0.035627163 | 61.20% | 38.665 | 12.23669 | 0.139 | -0.014 | 0.027 | 0.229 |
|  | HVEM on naive CD8br | 20.82935782 | 0.076373012 | 21.42770613 | 0.091163563 | 22.10% | 12.835 | 16.29466 | 0.319 | 0.011 | 0.02 | 0.079 |
|  | CD3- lymphocyte %lymphocyte | 18.44878551 | 0.732712002 | 18.44878636 | 0.780711223 | 0.00% | 14.464 | 13.25497 | 0.865 | -0.001 | 0.066 | 0.894 |
|  | CD28 on CD28+ DN (CD4-CD8-) | 15.86795494 | 0.601762357 | 16.42173608 | 0.628980461 | 0.00% | 11.467 | 16.96439 | 0.136 | NA | NA | NA |
| Cochran Q statistic implemented in MR Egger and IVW method, P>0.05 indicates no heterogeneity exists. The intercept of MR Egger can be used to indicate whether directional horizontal pleiotropy is driving the results of MR analysis, there are no directional pleiotropies if P>0.05.  MR-PRESSO can detect and adjust for any outliers reflecting horizontal pleiotropic biases, where p value for Global test > 0.05 indicates no horizontal pleiotropic outliers. The I2 statistic was calculated to assess the heterogeneity of each outcome from different data sources, and the I2values <25%，25-75%,and >75% were considered to indicate low moderate,and high heterogeneity, respectively. | | | | | | | | | | | | |

| **Supplementary materials Table 3. Reverse mendelian causality analysis of 731 immune cells and different lymphoma** | | | | | | | | | | | | | |
| --- | --- | --- | --- | --- | --- | --- | --- | --- | --- | --- | --- | --- | --- |
| **Exposure** | **ID.outcome** | **Immune cell** | **panel** | **Pleiotropy** | **Inverse variance weighted** | | | **Weighted median** | | | **MR Egger** | | |
|  |  |  |  |  | **b** | **se** | **pval** | **b** | **se** | **pval** | **b** | **se** | **pval** |
| Hodgkin lymphoma | GCST90001457 | CD11c+ HLA DR++ monocyte %monocyte | cDC | 0.062 | 0.021 | 0.011 | 0.065 | 0.016 | 0.012 | 0.198 | 0.007 | 0.011 | 0.536 |
|  | GCST90001625 | HLA DR+ CD4+ %T cell | TBNK | 0.112 | ＜0.001 | 0.014 | 0.969 | 0.008 | 0.009 | 0.396 | 0.015 | 0.016 | 0.347 |
|  | GCST90001626 | HLA DR+ CD4+ %lymphocyte | TBNK | 0.136 | ＜0.001 | 0.016 | 0.955 | 0.008 | 0.01 | 0.419 | 0.016 | 0.017 | 0.371 |
|  | GCST90001641 | CD3- lymphocyte %leukocyte | TBNK | 0.623 | 0.000 | 0.009 | 0.642 | ＜0.001 | 0.011 | 0.569 | ＜0.001 | 0.012 | 0.922 |
|  | GCST90001895 | CD28 on CD28+ DN (CD4-CD8-) | Treg | 0.301 | 0.006 | 0.009 | 0.517 | 0.001 | 0.011 | 0.874 | ＜0.001 | 0.011 | 0.959 |
|  | GCST90001729 | CD19 on IgD+ CD38br | B cell | 0.491 | ＜0.001 | 0.009 | 0.602 | ＜0.001 | 0.01 | 0.463 | ＜0.001 | 0.011 | 0.428 |
|  | GCST90001804 | CD27 on IgD- CD38dim | B cell | 0.392 | ＜0.001 | 0.009 | 0.976 | ＜0.001 | 0.01 | 0.582 | ＜0.001 | 0.011 | 0.652 |
|  | GCST90001838 | CD3 on naive CD8br | Maturation stages of T cell | 0.705 | -0.01 | 0.01 | 0.206 | -0.01 | 0.013 | 0.441 | -0.01 | 0.012 | 0.417 |
|  | GCST90001844 | CD3 on TD CD4+ | Maturation stages of T cell | 0.201 | 0.006 | 0.01 | 0.526 | 0.003 | 0.011 | 0.784 | ＜0.001 | 0.012 | 0.849 |
|  | GCST90001874 | HVEM on TD CD8br | Maturation stages of T cell | 0.148 | 0.004 | 0.014 | 0.738 | 0 | 0.016 | 0.988 | -0.01 | 0.017 | 0.582 |
|  | GCST90001941 | CD25 on secreting Treg | Treg | 0.68 | -0.01 | 0.009 | 0.273 | -0.01 | 0.012 | 0.227 | -0.01 | 0.011 | 0.279 |
|  | GCST90001989 | CD40 on CD14- CD16+ monocyte | Monocyte | 0.723 | 0.006 | 0.009 | 0.515 | 0.005 | 0.011 | 0.652 | 0.008 | 0.011 | 0.475 |
| Diffuse large B-cell lymphoma | GCST90001413 | IgD- CD24- %B cell | B cell | 0.424 | 0.000 | 0.015 | 0.974 | 0.003 | 0.020 | 0.862 | 0.019 | 0.026 | 0.501 |
|  | GCST90001596 | CD8dim AC | TBNK | 0.180 | -0.010 | 0.015 | 0.519 | 0.003 | 0.019 | 0.842 | 0.022 | 0.026 | 0.422 |
|  | GCST90001613 | DN (CD4-CD8-) %leukocyte | TBNK | 0.959 | -0.010 | 0.015 | 0.229 | -0.010 | 0.019 | 0.496 | -0.010 | 0.027 | 0.559 |
|  | GCST90001640 | CD3- lymphocyte %lymphocyte | TBNK | NA | -0.050 | 0.040 | 0.166 | NA | NA | NA | NA | NA | NA |
|  | GCST90001677 | CD28- CD25++ CD8br %CD8br | Treg | 0.645 | 0.017 | 0.018 | 0.345 | 0.005 | 0.019 | 0.779 | 0.003 | 0.034 | 0.914 |
|  | GCST90001687 | CD28- CD8br AC | Treg | 0.345 | 0.012 | 0.021 | 0.566 | 0.010 | 0.020 | 0.595 | 0.043 | 0.036 | 0.290 |
|  | GCST90001865 | CD3 on CD28- CD8br | Treg | 0.555 | -0.020 | 0.019 | 0.183 | -0.010 | 0.022 | 0.538 | 0.000 | 0.035 | 0.833 |
|  | GCST90001869 | CD3 on resting Treg | Treg | 0.349 | -0.020 | 0.018 | 0.251 | -0.020 | 0.023 | 0.377 | -0.040 | 0.031 | 0.191 |
|  | GCST90001870 | CD34 on HSC | Myeloid cell | 0.501 | -0.020 | 0.023 | 0.351 | 0.000 | 0.028 | 0.939 | 0.002 | 0.039 | 0.960 |
|  | GCST90001877 | HVEM on naive CD4+ | Maturation stages of T cell | 0.751 | -0.010 | 0.032 | 0.548 | -0.040 | 0.032 | 0.151 | -0.030 | 0.060 | 0.577 |
|  | GCST90001928 | CD127 on CD28+ CD45RA- CD8br | Treg | 0.508 | -0.030 | 0.022 | 0.097 | -0.010 | 0.022 | 0.469 | -0.010 | 0.039 | 0.744 |
|  | GCST90001941 | CD25 on secreting Treg | Treg | 0.568 | -0.010 | 0.016 | 0.240 | -0.010 | 0.021 | 0.400 | 0.000 | 0.028 | 0.861 |
|  | GCST90001989 | CD40 on CD14- CD16+ monocyte | Monocyte | 0.516 | -0.010 | 0.015 | 0.372 | -0.020 | 0.020 | 0.256 | -0.020 | 0.027 | 0.326 |
|  | GCST90002023 | CD4 on CM CD4 + | Maturation stages of T cell | 0.868 | -0.020 | 0.017 | 0.148 | -0.030 | 0.023 | 0.203 | -0.020 | 0.030 | 0.370 |
|  | GCST90002047 | CD45 on Gr MDSC | Myeloid cell | 0.933 | 0.019 | 0.026 | 0.461 | 0.018 | 0.033 | 0.584 | 0.016 | 0.050 | 0.763 |
|  | GCST90002073 | SSC-A on monocyte | cDC | 0.977 | 0.000 | 0.017 | 0.753 | 0.000 | 0.021 | 0.696 | 0.000 | 0.030 | 0.843 |
|  | GCST90002074 | SSC-A on CD14+ monocyte | TBNK | 0.609 | -0.010 | 0.016 | 0.249 | -0.010 | 0.021 | 0.344 | 0.000 | 0.028 | 0.825 |
|  | GCST90002110 | HLA DR on CD33dim HLA DR+ CD11b+ | Myeloid cell | 0.138 | 0.000 | 0.026 | 0.760 | -0.010 | 0.032 | 0.642 | 0.049 | 0.039 | 0.271 |
|  | GCST90002113 | HLA DR on HLA DR+ T cell | TBNK | 0.936 | 0.009 | 0.016 | 0.554 | 0.013 | 0.021 | 0.509 | 0.007 | 0.028 | 0.793 |
| mature T/NK-cell lymphomas | GCST90001413 | IgD- CD24- %B cell | B cell | 0.803 | 0.000 | 0.013 | 0.864 | -0.020 | 0.018 | 0.277 | 0.000 | 0.019 | 0.772 |
|  | GCST90001414 | IgD- CD24- AC | B cell | 0.911 | 0.000 | 0.017 | 0.975 | -0.030 | 0.019 | 0.081 | 0.000 | 0.027 | 0.920 |
|  | GCST90001420 | IgD- CD38br AC | B cell | 0.707 | -0.010 | 0.012 | 0.144 | -0.020 | 0.016 | 0.116 | -0.010 | 0.017 | 0.492 |
|  | GCST90001457 | CD11c+ HLA DR++ monocyte %monocyte | cDC | 0.511 | -0.010 | 0.013 | 0.336 | -0.010 | 0.018 | 0.542 | -0.020 | 0.019 | 0.286 |
|  | GCST90001734 | CD19 on IgD- CD38br | B cell | 0.029 | 0.000 | 0.018 | 0.915 | -0.020 | 0.020 | 0.170 | -0.040 | 0.018 | 0.076 |
|  | GCST90001774 | CD24 on transitional | B cell | 0.177 | -0.030 | 0.017 | 0.028 | -0.020 | 0.018 | 0.158 | -0.060 | 0.023 | 0.032 |
|  | GCST90001830 | BAFF-R on CD20- | B cell | 0.206 | 0.010 | 0.012 | 0.403 | 0.011 | 0.017 | 0.497 | 0.000 | 0.018 | 0.677 |
|  | GCST90001832 | CD62L on CD62L+ plasmacytoid DC | cDC | 0.509 | -0.030 | 0.013 | 0.015 | -0.020 | 0.018 | 0.208 | -0.020 | 0.020 | 0.302 |
|  | GCST90001838 | CD3 on naive CD8br | Maturation stages of T cell | 0.150 | 0.010 | 0.016 | 0.508 | 0.000 | 0.018 | 0.929 | -0.010 | 0.021 | 0.535 |
|  | GCST90001846 | CD3 on CM CD8br | Maturation stages of T cell | 0.327 | 0.003 | 0.017 | 0.858 | -0.010 | 0.018 | 0.452 | -0.010 | 0.024 | 0.550 |
|  | GCST90001864 | CD3 on CD28+ CD45RA+ CD8br | Treg | 0.180 | 0.007 | 0.016 | 0.641 | 0.000 | 0.019 | 0.962 | -0.010 | 0.020 | 0.495 |
|  | GCST90001865 | CD3 on CD28- CD8br | Treg | 0.605 | 0.000 | 0.014 | 0.944 | 0.009 | 0.018 | 0.615 | 0.000 | 0.019 | 0.677 |
|  | GCST90001868 | CD3 on CD4 Treg | Treg | 0.910 | -0.010 | 0.021 | 0.450 | -0.030 | 0.026 | 0.207 | -0.010 | 0.030 | 0.671 |
|  | GCST90001885 | CD16-CD56 on HLA DR+ NK | TBNK | 0.796 | 0.000 | 0.013 | 0.591 | 0.000 | 0.018 | 0.620 | -0.010 | 0.019 | 0.589 |
|  | GCST90001900 | CD28 on resting Treg | Treg | 0.156 | 0.000 | 0.014 | 0.757 | -0.010 | 0.018 | 0.460 | -0.020 | 0.019 | 0.215 |
| other and unspecified types of non Hodgkin | GCST90001399 | IgD- CD27- %B cell | B cell | 0.52 | -0.01 | 0.02 | 0.51 | -0.03 | 0.03 | 0.24 | -0.03 | 0.04 | 0.37 |
|  | GCST90001420 | IgD- CD38br AC | B cell | 0.863 | -0.03 | 0.02 | 0.14 | -0.03 | 0.03 | 0.26 | -0.02 | 0.03 | 0.44 |
|  | GCST90001543 | EM CD4+ %CD4+ | Maturation stages of T cell | 0.821 | 0.04 | 0.02 | 0.06 | 0.06 | 0.03 | 0.06 | 0.03 | 0.03 | 0.34 |
|  | GCST90001607 | CD8br %leukocyte | TBNK | 0.936 | 0.00 | 0.02 | 0.92 | 0.02 | 0.03 | 0.54 | 0.00 | 0.04 | 0.90 |
|  | GCST90001625 | HLA DR+ CD4+ %T cell | TBNK | 0.236 | 0.00 | 0.02 | 0.90 | -0.03 | 0.03 | 0.23 | -0.03 | 0.03 | 0.32 |
|  | GCST90001630 | CD8br NKT AC | TBNK | 0.684 | 0.01 | 0.03 | 0.77 | 0.03 | 0.03 | 0.35 | 0.02 | 0.04 | 0.63 |
|  | GCST90001677 | CD28- CD25++ CD8br %CD8br | Treg | 0.358 | 0.02 | 0.02 | 0.33 | 0.04 | 0.03 | 0.14 | 0.05 | 0.03 | 0.20 |
|  | GCST90001678 | CD28- CD25++ CD8br AC | Treg | 0.295 | 0.02 | 0.03 | 0.49 | 0.06 | 0.03 | 0.07 | 0.05 | 0.04 | 0.23 |
|  | GCST90001679 | CD25++ CD8br %T cell | Treg | 0.972 | 0.01 | 0.02 | 0.77 | 0.00 | 0.03 | 0.94 | 0.01 | 0.04 | 0.89 |
|  | GCST90001682 | CD127- CD8br %T cell | Treg | 0.991 | 0.00 | 0.02 | 0.91 | 0.01 | 0.03 | 0.66 | 0.00 | 0.04 | 0.95 |
|  | GCST90001687 | CD28- CD8br AC | Treg | 0.523 | 0.01 | 0.03 | 0.77 | 0.05 | 0.03 | 0.14 | 0.03 | 0.05 | 0.51 |
|  | GCST90001714 | BAFF-R on IgD- CD38dim | B cell | 0.718 | 0.06 | 0.05 | 0.21 | 0.05 | 0.06 | 0.34 | 0.10 | 0.09 | 0.48 |
|  | GCST90001729 | CD19 on IgD+ CD38br | B cell | 0.744 | 0.02 | 0.02 | 0.28 | 0.01 | 0.03 | 0.66 | 0.02 | 0.04 | 0.67 |
|  | GCST90001774 | CD24 on transitional | B cell | 0.843 | 0.01 | 0.02 | 0.68 | 0.01 | 0.03 | 0.76 | 0.01 | 0.03 | 0.69 |
|  | GCST90001826 | IgD on unsw mem | B cell | 0.43 | 0.00 | 0.02 | 0.89 | 0.00 | 0.03 | 0.94 | -0.02 | 0.04 | 0.60 |
|  | GCST90001827 | IgD on IgD+ | B cell | 0.647 | 0.03 | 0.02 | 0.27 | 0.01 | 0.03 | 0.67 | 0.01 | 0.04 | 0.74 |
|  | GCST90001870 | CD34 on HSC | Myeloid cell | 0.842 | -0.03 | 0.05 | 0.42 | -0.01 | 0.05 | 0.81 | -0.02 | 0.08 | 0.75 |
|  | GCST90001877 | HVEM on naive CD4+ | Maturation stages of T cell | 0.795 | -0.03 | 0.04 | 0.40 | -0.02 | 0.05 | 0.57 | -0.01 | 0.06 | 0.76 |
|  | GCST90002074 | SSC-A on CD14+ monocyte | TBNK | 0.732 | -0.03 | 0.03 | 0.25 | -0.01 | 0.03 | 0.61 | -0.01 | 0.04 | 0.68 |
| follicular lymphoma | GCST90001607 | CD8br %leukocyte | TBNK | 0.429 | 0.039 | 0.025 | 0.124 | 0.026 | 0.027 | 0.345 | 0.005 | 0.049 | 0.913 |
|  | GCST90001640 | CD3- lymphocyte %lymphocyte | TBNK | 0.844 | -0.040 | 0.024 | 0.072 | -0.010 | 0.028 | 0.531 | -0.050 | 0.049 | 0.332 |
|  | GCST90001677 | CD28- CD25++ CD8br %CD8br | Treg | 0.877 | 0.000 | 0.020 | 0.712 | 0.000 | 0.027 | 0.986 | 0.000 | 0.039 | 0.958 |
|  | GCST90001678 | CD28- CD25++ CD8br AC | Treg | 0.858 | 0.022 | 0.023 | 0.337 | 0.018 | 0.029 | 0.539 | 0.029 | 0.046 | 0.534 |
|  | GCST90001682 | CD127- CD8br %T cell | Treg | 0.815 | 0.029 | 0.025 | 0.249 | 0.018 | 0.029 | 0.543 | 0.019 | 0.050 | 0.709 |
|  | GCST90001734 | CD19 on IgD- CD38br | B cell | 0.254 | 0.031 | 0.023 | 0.194 | 0.016 | 0.028 | 0.551 | -0.010 | 0.043 | 0.759 |
|  | GCST90001749 | CD20 on IgD+ CD38- naive | B cell | 0.366 | 0.036 | 0.026 | 0.177 | 0.049 | 0.036 | 0.171 | 0.076 | 0.050 | 0.160 |
|  | GCST90001814 | CD38 on IgD- CD38br | B cell | 0.756 | -0.010 | 0.021 | 0.640 | 0.004 | 0.027 | 0.871 | 0.001 | 0.042 | 0.975 |
|  | GCST90001839 | CD3 on EM CD8br | Maturation stages of T cell | 0.824 | 0.000 | 0.021 | 0.935 | 0.000 | 0.030 | 0.847 | 0.000 | 0.040 | 0.818 |
|  | GCST90001844 | CD3 on TD CD4+ | Maturation stages of T cell | 0.589 | -0.040 | 0.021 | 0.050 | -0.040 | 0.032 | 0.126 | -0.060 | 0.040 | 0.161 |
|  | GCST90001872 | HVEM on naive CD8br | Maturation stages of T cell | 0.133 | 0.030 | 0.032 | 0.345 | 0.008 | 0.045 | 0.854 | -0.050 | 0.059 | 0.406 |
|  | GCST90001895 | CD28 on CD28+ DN (CD4-CD8-) | Treg | NA | 0.127 | 0.066 | 0.055 | NA | NA | NA | NA | NA | NA |
|  | GCST90001988 | HLA DR on CD14+ CD16- monocyte | Monocyte | 0.858 | 0.053 | 0.043 | 0.211 | 0.002 | 0.028 | 0.930 | 0.040 | 0.083 | 0.637 |
|  | GCST90002038 | CD80 on CD62L+ plasmacytoid DC | cDC | 0.257 | -0.020 | 0.035 | 0.536 | -0.060 | 0.034 | 0.072 | -0.080 | 0.065 | 0.205 |
|  | GCST90002056 | CD8 on EM CD8br | Maturation stages of T cell | 0.466 | 0.016 | 0.023 | 0.493 | 0.005 | 0.029 | 0.845 | -0.010 | 0.044 | 0.783 |
|  | GCST90002073 | SSC-A on monocyte | cDC | 0.332 | -0.010 | 0.021 | 0.542 | -0.020 | 0.031 | 0.431 | -0.040 | 0.040 | 0.262 |
| non-follicular lymphoma | GCST90001457 | CD11c+ HLA DR++ monocyte %monocyte | cDC | 0.642 | 0.002 | 0.062 | 0.968 | 0.086 | 0.071 | 0.229 | 0.108 | 0.227 | 0.649 |
|  | GCST90001592 | CD8br AC | TBNK | 0.953 | -0.060 | 0.048 | 0.169 | 0.000 | 0.059 | 0.926 | -0.050 | 0.184 | 0.769 |
|  | GCST90001607 | CD8br %leukocyte | TBNK | 0.857 | -0.080 | 0.045 | 0.073 | -0.040 | 0.058 | 0.412 | -0.050 | 0.156 | 0.749 |
|  | GCST90001630 | CD8br NKT AC | TBNK | 0.839 | -0.070 | 0.054 | 0.174 | -0.070 | 0.062 | 0.233 | -0.030 | 0.203 | 0.878 |
|  | GCST90001677 | CD28- CD25++ CD8br %CD8br | Treg | 0.404 | -0.070 | 0.074 | 0.315 | -0.030 | 0.064 | 0.586 | 0.154 | 0.267 | 0.583 |
|  | GCST90001729 | CD19 on IgD+ CD38br | B cell | 0.449 | -0.110 | 0.056 | 0.043 | -0.050 | 0.066 | 0.394 | 0.041 | 0.202 | 0.842 |
|  | GCST90001824 | IgD on IgD+ CD38br | B cell | 0.856 | 0.032 | 0.045 | 0.468 | 0.067 | 0.059 | 0.255 | 0.060 | 0.155 | 0.709 |
|  | GCST90001826 | IgD on unsw mem | B cell | 0.728 | 0.066 | 0.060 | 0.271 | 0.049 | 0.061 | 0.426 | 0.144 | 0.222 | 0.541 |
|  | GCST90001827 | IgD on IgD+ | B cell | 0.921 | 0.064 | 0.045 | 0.156 | 0.089 | 0.061 | 0.146 | 0.080 | 0.157 | 0.628 |
|  | GCST90001867 | CD3 on CD4+ | Treg | 0.624 | 0.056 | 0.057 | 0.323 | 0.002 | 0.066 | 0.975 | -0.040 | 0.210 | 0.830 |
|  | GCST90001870 | CD34 on HSC | Myeloid cell | 0.866 | 0.123 | 0.069 | 0.075 | 0.182 | 0.091 | 0.045 | 0.080 | 0.255 | 0.764 |
|  | GCST90001900 | CD28 on resting Treg | Treg | 0.205 | -0.020 | 0.085 | 0.776 | -0.070 | 0.085 | 0.353 | -0.370 | 0.199 | 0.203 |
|  | GCST90001941 | CD25 on secreting Treg | Treg | 0.665 | 0.007 | 0.046 | 0.871 | 0.008 | 0.059 | 0.890 | -0.060 | 0.163 | 0.710 |
|  | GCST90002062 | CD4 on CD28+ CD4+ | Treg | 0.821 | 0.039 | 0.066 | 0.545 | 0.078 | 0.070 | 0.264 | 0.095 | 0.247 | 0.711 |
|  | GCST90002074 | SSC-A on CD14+ monocyte | TBNK | 0.838 | 0.023 | 0.048 | 0.632 | 0.072 | 0.066 | 0.274 | 0.059 | 0.176 | 0.748 |
|  | GCST90002106 | HLA DR on DC | cDC | 0.899 | -0.220 | 0.200 | 0.254 | -0.050 | 0.070 | 0.420 | -0.130 | 0.751 | 0.864 |
|  | GCST90002110 | HLA DR on CD33dim HLA DR+ CD11b+ | Myeloid cell | 0.891 | -0.170 | 0.100 | 0.076 | -0.110 | 0.087 | 0.174 | -0.120 | 0.369 | 0.739 |
|  | GCST90002112 | HLA DR on CD33- HLA DR+ | Myeloid cell | 0.684 | -0.150 | 0.176 | 0.376 | 0.000 | 0.093 | 0.923 | 0.104 | 0.640 | 0.875 |
| Reverse mendelian analysis of different lymphomas and immune cells | | | | | | | | | | | | | |

| **Supplementary materials Table 4. LDSC results of diverse immune cells causally associated with different lymphoma** | | | | | | |
| --- | --- | --- | --- | --- | --- | --- |
| **Immune cells** | **disease** | **Genetic correlation** | | | | |
|  |  | **Genetic correlation** | **SE** | P value for LDSC | **Intercept** | **Intercept (SE)** |
| IgD- CD24- %B cell | Mature T/NK-cell lymphomas | -2.06E-01 | 3.57E-01 | 0.4873 | 3.50E-03 | 4.10E-03 |
|  | Diffuse large B-cell lymphoma | -3.32E-01 | 1.22E-01 | 0.3316 | -8.80E-03 | 6.10E-03 |
| IgD- CD38br AC | Other and unspecified types of non-Hodgkin | 1.12E-01 | 7.67E-01 | 0.8834 | -5.40E-03 | 4.30E-03 |
|  | Mature T/NK-cell lymphomas | 2.64E-01 | 2.48E-01 | 0.0873 | -6.00E-04 | 4.00E-03 |
| CD11c+ HLA DR++ monocyte %monocyte | Hodgkin lymphoma | -4.12E-01 | 2.59E-01 | 0.0223 | 2.60E-03 | 3.80E-03 |
|  | Non-follicular lymphoma | 3.39E-01 | 2.40E-01 | 0.1873 | -2.20E-03 | 4.50E-03 |
|  | Mature T/NK-cell lymphomas | -3.06E-01 | 1.24E-01 | 0.0187 | 8.00E-04 | 3.00E-04 |
| CD8br %leukocyte | Other and unspecified types of non-Hodgkin | 4.06E-01 | 2.75E-01 | 0.0224 | -3.00E-04 | -2.00E-03 |
|  | Follicular lymphoma | 3.06E-01 | 1.25E-01 | 0.0173 | -4.10E-03 | 2.00E-04 |
|  | Non-follicular lymphoma | 3.95E-01 | 1.58E-01 | 0.02873 | -3.00E-04 | 4.00E-03 |
| HLA DR+ CD4+ %T cell | Hodgkin lymphoma | -3.06E-01 | 2.12E-01 | 0.0473 | -1.30E-03 | 9.00E-04 |
|  | Other and unspecified types of non-Hodgkin | -2.06E-01 | 7.48E-02 | 0.0125 | -4.30E-04 | 5.00E-05 |
| CD8br NKT AC | Other and unspecified types of non-Hodgkin | 3.16E-01 | 1.46E-01 | 0.0216 | -2.50E-03 | 8.50E-04 |
|  | Non-follicular lymphoma | 4.06E-01 | 3.48E-01 | 0.0682 | -2.50E-03 | 4.10E-03 |
| CD3- lymphocyte %lymphocyte | Follicular lymphoma | -2.35E-01 | 4.96E-01 | 0.3873 | -7.50E-03 | 4.40E-03 |
|  | Diffuse large B-cell lymphoma | 1.17E-01 | 2.27E-01 | 0.5482 | 2.00E-03 | 4.30E-03 |
| CD28- CD25++ CD8br %CD8br | Other and unspecified types of non-Hodgkin | 4.16E-01 | 2.96E-01 | 0.0343 | 2.10E-03 | 4.10E-04 |
|  | Follicular lymphoma | 3.27E-01 | 1.62E-01 | 0.0294 | -4.00E-04 | 3.90E-04 |
|  | Non-follicular lymphoma | 4.46E-01 | 2.95E-01 | 0.0306 | 2.10E-03 | 1.20E-03 |
|  | Diffuse large B-cell lymphoma | 3.94E-01 | 2.02E-01 | 0.0247 | -2.50E-04 | 1.50E-04 |
| CD28- CD25++ CD8br AC | Other and unspecified types of non-Hodgkin | 5.23E-01 | 7.64E-01 | 0.6273 | 6.00E-04 | 4.00E-03 |
|  | Follicular lymphoma | 3.42E-01 | 1.39E-01 | 0.0192 | -1.50E-03 | 8.40E-04 |
| CD127- CD8br %T cell | Other and unspecified types of non-Hodgkin | 2.97E-01 | 1.13E-01 | 0.0393 | -1.40E-03 | 1.21E-04 |
|  | Follicular lymphoma | 5.82E-01 | 3.27E-01 | 0.0402 | -6.90E-03 | 4.10E-03 |
| CD28- CD8br AC | Other and unspecified types of non-Hodgkin | 2.12E-01 | 2.93E-01 | 0.3947 | -3.45E-06 | 4.00E-03 |
|  | Diffuse large B-cell lymphoma | 3.24E-01 | 5.92E-01 | 0.6297 | -4.50E-03 | 4.20E-03 |
| CD19 on IgD+ CD38br | Hodgkin lymphoma | 4.28E-01 | 2.07E-01 | 0.0029 | 5.50E-03 | 6.20E-04 |
|  | Other and unspecified types of non-Hodgkin | 3.27E-01 | 3.62E-01 | 0.3674 | -4.00E-04 | 3.90E-03 |
|  | Non-follicular lymphoma | 3.85E-01 | 3.92E-01 | 0.4064 | -3.00E-04 | 3.50E-03 |
| CD19 on IgD- CD38br | Follicular lymphoma | -5.92E-01 | 3.95E-01 | 0.0343 | -6.00E-04 | 1.40E-04 |
|  | Mature T/NK-cell lymphomas | -2.64E-01 | 1.10E-01 | 0.0393 | 2.10E-03 | 2.30E-04 |
| CD24 on transitional | Other and unspecified types of non-Hodgkin | -2.36E-01 | 2.36E-01 | 0.3681 | -2.90E-03 | 3.90E-03 |
|  | Mature T/NK-cell lymphomas | 3.24E-01 | 5.92E-01 | 0.3682 | -3.40E-03 | 3.40E-03 |
| IgD on unsw mem | Other and unspecified types of non-Hodgkin | -3.95E-01 | 2.40E-01 | 0.01963 | 2.10E-03 | 7.20E-04 |
|  | Non-follicular lymphoma | -3.85E-01 | 1.93E-01 | 0.01563 | 4.50E-04 | 1.30E-04 |
| IgD on IgD+ | Other and unspecified types of non-Hodgkin | -3.93E-01 | 1.94E-01 | 0.0283 | 3.50E-04 | 4.80E-04 |
|  | Non-follicular lymphoma | -3.96E-01 | 4.79E-01 | 0.4283 | 1.00E-03 | 3.80E-03 |
| CD3 on TD CD4+ | Hodgkin lymphoma | -3.92E-01 | 1.12E+00 | 0.7264 | 2.20E-03 | 4.50E-03 |
|  | Follicular lymphoma | 5.62E-01 | 3.97E-01 | 0.0937 | -3.00E-04 | 2.00E-03 |
| CD3 on CD28- CD8br | Mature T/NK-cell lymphomas | -2.58E-01 | 9.11E-02 | 0.0182 | 2.80E-03 | 2.70E-03 |
|  | Diffuse large B-cell lymphoma | -3.38E-01 | 1.99E-01 | 0.0273 | 4.00E-04 | 1.70E-04 |
| CD34 on HSC | Other and unspecified types of non-Hodgkin | 2.42E-01 | 3.36E-01 | 0.1363 | 6.50E-03 | 1.23E-03 |
|  | Non-follicular lymphoma | 1.06E-01 | 7.48E-01 | 0.8873 | 3.10E-03 | 3.90E-03 |
|  | Diffuse large B-cell lymphoma | -1.06E-01 | 6.73E-01 | 0.8748 | -1.70E-03 | 3.90E-03 |
| HVEM on naive CD4+ | Other and unspecified types of non-Hodgkin | -2.36E-01 | 1.11E-01 | 0.0382 | 3.80E-04 | 1.70E-04 |
|  | Diffuse large B-cell lymphoma | -4.95E-01 | 2.42E-01 | 0.01093 | 2.30E-03 | 1.60E-04 |
| CD28 on resting Treg | Non-follicular lymphoma | 4.74E-01 | 4.10E-01 | 0.1934 | 5.50E-04 | 2.30E-04 |
|  | Mature T/NK-cell lymphomas | 1.27E-01 | 2.48E-01 | 0.3048 | 3.20E-04 | 4.50E-03 |
| CD25 on secreting Treg | Hodgkin lymphoma | 4.52E-01 | 3.02E-01 | 0.0406 | 4.20E-04 | 2.30E-05 |
|  | Non-follicular lymphoma | 5.24E-01 | 3.91E-01 | 0.0347 | -6.90E-03 | 4.20E-04 |
|  | Diffuse large B-cell lymphoma | 5.21E-01 | 3.45E-01 | 0.0231 | 3.00E-04 | 3.20E-04 |
| CD40 on CD14- CD16+ monocyte | Hodgkin lymphoma | 1.93E-01 | 2.79E-01 | 0.2353 | 3.50E-03 | 4.50E-03 |
|  | Diffuse large B-cell lymphoma | -1.20E-01 | 6.79E-01 | 0.7349 | -5.30E-03 | 3.50E-04 |
| SSC-A on monocyte | Follicular lymphoma | 3.35E-01 | 2.60E-01 | 0.1526 | 2.00E-03 | 3.90E-03 |
|  | Diffuse large B-cell lymphoma | 4.08E-01 | 3.40E-01 | 0.0924 | 4.00E-03 | 4.40E-03 |
| SSC-A on CD14+ monocyte | Other and unspecified types of non-Hodgkin | -3.95E-01 | 5.28E-01 | 0.4974 | 2.30E-03 | 5.20E-04 |
|  | Non-follicular lymphoma | 3.21E-01 | 5.38E-01 | 0.5506 | 1.40E-03 | 4.30E-03 |
|  | Diffuse large B-cell lymphoma | 6.32E-01 | 3.22E-01 | 0.0196 | -4.80E-03 | 4.60E-04 |
| HLA DR on CD33dim HLA DR+ CD11b+ | Non-follicular lymphoma | -3.59E-01 | 3.41E-01 | 0.0783 | -5.90E-03 | 3.90E-03 |
|  | Diffuse large B-cell lymphoma | -3.48E-01 | 2.60E-01 | 0.0462 | 7.50E-03 | 3.70E-04 |

| **Supplementary materials Table 5. SMR results of diverse immune cells causally associated with different lymphoma** | | | | | | | | | | | | | | | | | | | | | | | |  |
| --- | --- | --- | --- | --- | --- | --- | --- | --- | --- | --- | --- | --- | --- | --- | --- | --- | --- | --- | --- | --- | --- | --- | --- | --- |
| **Type of immune cells** | **Probe** | **Gene Chr.** | **Gene** | **Probe base pair** | **topSNP** | **Gene Chr.** | **Probe base pair** | **Effect allele** | **Other allele** | **Effect allele frequence** | **GWAS association** | | | **eQTL association** | | | **SMR association** | | | **HEIDI Test** | | ***PFDR*** | ***PP.H4*** | |
|  |  |  |  |  |  |  |  |  |  |  | **β** | **SE** | ***P*** | **β** | **SE** | ***P*** | **β** | **SE** | ***P*** | ***P*** | **nsnp** |  |  |  |
| IgD- CD24- %B cell | ENSG00000226278 | 7 | PSPHP1 | 55832490 | rs6972291 | 7 | 55802063 | C | T | 0.234592 | 1.09E-01 | 2.97E-02 | 2.68E-04 | 1.05E+00 | 8.82E-02 | 6.55E-33 | 1.03E-01 | 2.95E-02 | 4.82E-04 | 4.69E-01 | 2.00E+01 | 1.86E-02 | 0.00826313 | |
|  | ENSG00000185290 | 7 | NUPR1L | 56182374 | rs10232851 | 7 | 55828834 | A | G | 0.234592 | 1.09E-01 | 2.97E-02 | 2.55E-04 | 1.00E+00 | 8.70E-02 | 1.20E-30 | 1.09E-01 | 3.11E-02 | 4.84E-04 | 7.05E-01 | 2.00E+01 | 1.86E-02 | 0.183743 | |
| CD11c+ HLA DR++ monocyte %monocyte | ENSG00000204149 | 10 | AGAP6 | 51748078 | rs4463775 | 10 | 51128803 | A | G | 0.331014 | -1.05E-01 | 3.06E-02 | 5.91E-04 | -5.41E-01 | 8.10E-02 | 2.41E-11 | 1.94E-01 | 6.35E-02 | 1.23E-04 | 1.85E-01 | 7.00E+00 | 2.00E-02 | 0.01823674 | |
| CD8br %leukocyte | ENSG00000198502 | 6 | HLA-DRB5 | 32485120 | rs71549223 | 6 | 32498094 | A | G | 0.83499 | 1.88E-01 | 4.75E-02 | 7.99E-05 | -1.03E+00 | 1.21E-01 | 1.45E-17 | -1.82E-01 | 5.08E-02 | 3.40E-04 | 6.07E-02 | 2.00E+01 | 1.99E-02 | 0.2937463 | |
|  | ENSG00000110665 | 11 | C11orf21 | 2316875 | rs734094 | 11 | 2323220 | A | G | 0.399602 | -9.15E-02 | 2.46E-02 | 2.06E-04 | -1.01E+00 | 1.11E-01 | 5.06E-20 | 9.03E-02 | 2.62E-02 | 5.75E-04 | 8.45E-01 | 1.60E+01 | 2.98E-02 | 0.017264 | |
|  | ENSG00000064201 | 11 | TSPAN32 | 2323227 | rs734094 | 11 | 2323220 | A | G | 0.399602 | -9.15E-02 | 2.46E-02 | 2.06E-04 | -9.00E-01 | 1.14E-01 | 2.38E-15 | 1.02E-01 | 3.02E-02 | 7.69E-04 | 6.37E-01 | 1.60E+01 | 3.98E-02 | 0.86836335 | |
| EM CD4+ %CD4+ | ENSG00000196301 | 6 | HLA-DRB9 | 32427598 | rs72844187 | 6 | 32521419 | G | C | 0.368787 | -2.26E-01 | 5.54E-02 | 4.71E-05 | 1.01E+00 | 9.99E-02 | 7.78E-24 | -2.24E-01 | 5.94E-02 | 1.58E-04 | 1.54E-01 | 2.00E+01 | 1.57E-01 | 0.87643836 | |
|  | ENSG00000198502 | 6 | HLA-DRB5 | 32485120 | rs71549223 | 6 | 32498094 | A | G | 0.83499 | -2.27E-01 | 4.77E-02 | 2.07E-06 | -1.03E+00 | 1.21E-01 | 1.45E-17 | 2.20E-01 | 5.30E-02 | 3.28E-05 | 1.40E-02 | 2.00E+01 | 6.49E-02 | 0.867128363 | |
| CD8br AC | ENSG00000198502 | 6 | HLA-DRB5 | 32485120 | rs71549223 | 6 | 32498094 | A | G | 0.83499 | 2.62E-01 | 4.57E-02 | 1.05E-08 | -1.03E+00 | 1.21E-01 | 1.45E-17 | -2.55E-01 | 5.35E-02 | 1.94E-06 | 1.10E-01 | 2.00E+01 | 3.83E-03 | 0.734628364 | |
| HLA DR+ CD4+ %T cell | ENSG00000104835 | 19 | SARS2 | 39405906 | rs730078 | 19 | 39421388 | G | A | 0.668986 | -1.05E-01 | 2.35E-02 | 7.85E-06 | -6.47E-01 | 9.48E-02 | 8.95E-12 | 1.62E-01 | 4.34E-02 | 1.83E-05 | 8.38E-01 | 1.10E+01 | 3.63E-02 | 0.07562984 | |
| CD8br NKT AC | ENSG00000110665 | 11 | C11orf21 | 2316875 | rs734094 | 11 | 2323220 | A | G | 0.399602 | -1.12E-01 | 2.49E-02 | 7.52E-06 | -1.01E+00 | 1.11E-01 | 5.06E-20 | 1.10E-01 | 2.74E-02 | 5.61E-05 | 7.05E-01 | 1.60E+01 | 3.41E-02 | 0.008471985 | |
|  | ENSG00000064201 | 11 | TSPAN32 | 2323227 | rs734094 | 11 | 2323220 | A | G | 0.399602 | -1.12E-01 | 2.49E-02 | 7.52E-06 | -9.00E-01 | 1.14E-01 | 2.38E-15 | 1.24E-01 | 3.18E-02 | 9.50E-05 | 6.66E-01 | 1.60E+01 | 3.41E-02 | 0.89477194 | |
| CD28- CD25++ CD8br %CD8br | ENSG00000116874 | 1 | WARS2 | 119573839 | rs12086 | 1 | 119573860 | C | T | 0.254473 | 1.06E-01 | 2.86E-02 | 2.32E-04 | -7.82E-01 | 9.89E-02 | 2.54E-15 | -1.35E-01 | 4.04E-02 | 8.37E-04 | 7.68E-01 | 2.00E+01 | 2.65E-02 | 0.792373613 | |
|  | ENSG00000143851 | 1 | PTPN7 | 202116141 | rs4309039 | 1 | 202129826 | C | T | 0.434394 | -9.94E-02 | 2.49E-02 | 6.82E-05 | -5.10E-01 | 8.71E-02 | 4.72E-09 | 1.95E-01 | 5.91E-02 | 9.81E-04 | 7.00E-01 | 5.00E+00 | 2.65E-02 | 0.781746513 | |
| CD28- CD8br AC | ENSG00000198502 | 6 | HLA-DRB5 | 32485120 | rs71549223 | 6 | 32498094 | A | G | 0.83499 | 2.26E-01 | 4.82E-02 | 2.90E-06 | -1.03E+00 | 1.21E-01 | 1.45E-17 | -2.19E-01 | 5.34E-02 | 4.00E-05 | 6.38E-02 | 2.00E+01 | 2.91E-02 | 0.7193753 | |
| BAFF-R on IgD- CD38dim | ENSG00000100395 | 22 | L3MBTL2 | 41601209 | rs139497 | 22 | 41640098 | T | C | 0.692843 | 3.07E-01 | 2.56E-02 | 1.52E-32 | -2.41E-01 | 4.24E-02 | 1.28E-08 | -1.27E+00 | 2.48E-01 | 2.74E-07 | 7.34E-02 | 4.00E+00 | 1.36E-04 | 0.779671534 | |
|  | ENSG00000235513 | 22 | RP4-756G23.5 | 41605126 | rs11913442 | 22 | 41593581 | T | C | 0.634195 | -1.95E-01 | 2.74E-02 | 1.36E-12 | 6.73E-01 | 9.24E-02 | 3.28E-13 | -2.89E-01 | 5.69E-02 | 3.59E-07 | 9.48E-02 | 1.10E+01 | 1.42E-04 | 0.208571843 | |
|  | ENSG00000184983 | 22 | NDUFA6 | 42481529 | rs117529804 | 22 | 42669104 | T | C | 0.028827 | 3.58E-01 | 5.30E-02 | 1.61E-11 | 1.77E+00 | 1.97E-01 | 2.37E-19 | 2.03E-01 | 3.75E-02 | 6.53E-08 | 7.36E-02 | 8.00E+00 | 4.31E-05 | 0.7741834 | |
| CD20 on IgD+ CD38- naive | ENSG00000228782 | 17 | MRPL45P2 | 45527934 | rs9894179 | 17 | 45565093 | T | C | 0.517893 | -1.43E-01 | 3.34E-02 | 2.07E-05 | 9.93E-01 | 8.85E-02 | 3.06E-29 | -1.44E-01 | 3.60E-02 | 6.61E-05 | 1.42E-01 | 2.00E+01 | 4.36E-02 | 0.102874683 | |
|  | ENSG00000196126 | 6 | HLA-DRB1 | 32546546 | rs74290599 | 6 | 32518785 | T | A | 0.327038 | -3.32E-01 | 6.60E-02 | 5.49E-07 | 8.05E-01 | 9.23E-02 | 2.81E-18 | -4.12E-01 | 9.47E-02 | 1.34E-05 | 5.30E-02 | 2.00E+01 | 1.32E-02 | 0.57166834 | |
|  | ENSG00000196301 | 6 | HLA-DRB9 | 32427598 | rs72844187 | 6 | 32521419 | G | C | 0.368787 | -3.44E-01 | 6.89E-02 | 6.26E-07 | 1.01E+00 | 9.99E-02 | 7.78E-24 | -3.42E-01 | 7.64E-02 | 7.52E-06 | 9.33E-02 | 2.00E+01 | 1.32E-02 | 0.018376746 | |
| IgD on IgD+ CD38br | ENSG00000128891 | 15 | C15orf57 | 40820882 | rs1044474 | 15 | 40845952 | G | A | 0.480119 | -1.65E-01 | 2.48E-02 | 3.00E-11 | -8.92E-01 | 9.73E-02 | 4.74E-20 | 1.85E-01 | 3.44E-02 | 6.99E-08 | 1.07E-01 | 2.00E+01 | 1.38E-04 | 0.7738165 | |
| IgD on unsw mem | ENSG00000137411 | 6 | VARS2 | 30876019 | rs2532934 | 6 | 30894759 | A | G | 0.558648 | -1.32E-01 | 2.82E-02 | 2.96E-06 | 5.42E-01 | 6.25E-02 | 4.36E-18 | -2.44E-01 | 5.93E-02 | 3.80E-05 | 6.11E-02 | 1.90E+01 | 2.51E-02 | 0.018232648 | |
| IgD on IgD+ CD38br | ENSG00000128891 | 15 | C15orf57 | 40820882 | rs1044474 | 15 | 40845952 | G | A | 0.480119 | -1.74E-01 | 2.52E-02 | 5.02E-12 | -8.92E-01 | 9.73E-02 | 4.74E-20 | 1.95E-01 | 3.53E-02 | 3.22E-08 | 6.51E-01 | 2.00E+01 | 6.38E-05 | 0.27461816 | |
|  | ENSG00000230658 | 7 | KLHL7-AS1 | 23140847 | rs13438452 | 7 | 23174182 | G | A | 0.345924 | 9.80E-02 | 2.54E-02 | 1.14E-04 | 1.14E+00 | 8.55E-02 | 1.23E-40 | 8.59E-02 | 2.32E-02 | 2.07E-04 | 2.81E-01 | 2.00E+01 | 1.36E-02 | 0.003878462 | |
|  | ENSG00000223313 | 15 | RNU6-516P | 40821769 | rs3803357 | 15 | 40751555 | A | C | 0.474155 | 1.35E-01 | 2.50E-02 | 8.18E-08 | 6.38E-01 | 1.10E-01 | 6.18E-09 | 2.11E-01 | 5.35E-02 | 7.94E-05 | 2.47E-01 | 6.00E+00 | 7.86E-03 | 0.2688693 | |
| BAFF-R on CD20- | ENSG00000183172 | 22 | SMDT1 | 42475695 | rs6002592 | 22 | 42476754 | C | A | 0.337972 | -1.03E-01 | 2.53E-02 | 4.98E-05 | -9.67E-01 | 8.77E-02 | 2.84E-28 | 1.06E-01 | 2.79E-02 | 1.38E-05 | 8.03E-02 | 2.00E+01 | 1.29E-02 | 0.1638976 | |
| CD3 on naive CD8br | ENSG00000086504 | 16 | MRPL28 | 417384 | rs3743884 | 16 | 420678 | T | C | 0.523857 | -1.49E-01 | 2.87E-02 | 2.22E-07 | -5.85E-01 | 5.96E-02 | 9.35E-23 | 2.55E-01 | 5.56E-02 | 4.39E-06 | 7.55E-02 | 2.00E+01 | 8.69E-03 | 0.1184683 | |
| CD3 on TD CD4+ | ENSG00000010626 | 12 | LRRC23 | 6982733 | rs66621175 | 12 | 6965805 | C | T | 0.318091 | 1.47E-01 | 3.04E-02 | 1.46E-06 | 7.66E-01 | 8.37E-02 | 5.35E-20 | 1.92E-01 | 4.49E-02 | 1.96E-05 | NA | NA | 3.83E-02 | 0.2295781 | |
| CD3 on CD28+ CD45RA+ CD8br | ENSG00000086504 | 16 | MRPL28 | 417384 | rs3743884 | 16 | 420678 | T | C | 0.523857 | -1.45E-01 | 2.84E-02 | 3.81E-07 | -5.85E-01 | 5.96E-02 | 9.35E-23 | 2.47E-01 | 5.47E-02 | 6.19E-06 | 2.15E-01 | 2.00E+01 | 1.22E-02 | 0.000384613 | |
| CD3 on CD4+ | ENSG00000196301 | 6 | HLA-DRB9 | 32427598 | rs72844187 | 6 | 32521419 | G | C | 0.368787 | 2.23E-01 | 5.68E-02 | 8.97E-05 | 1.01E+00 | 9.99E-02 | 7.78E-24 | 2.21E-01 | 6.06E-02 | 2.58E-05 | 6.89E-02 | 2.00E+01 | 2.74E-02 | 0.0637633 | |
|  | ENSG00000196126 | 6 | HLA-DRB1 | 32546546 | rs74290599 | 6 | 32518785 | T | A | 0.327038 | 2.18E-01 | 5.45E-02 | 6.51E-05 | 8.05E-01 | 9.23E-02 | 2.81E-18 | 2.71E-01 | 7.45E-02 | 2.77E-05 | 1.29E-01 | 2.00E+01 | 2.74E-02 | 0.5619469 | |
| HVEM on naive CD4+ | ENSG00000157873 | 1 | TNFRSF14 | 2487078 | rs1886730 | 1 | 2488608 | C | T | 0.481113 | 2.45E-01 | 3.80E-02 | 1.75E-10 | 2.40E-01 | 3.77E-02 | 1.92E-10 | 1.02E+00 | 2.25E-01 | 5.98E-06 | 9.39E-01 | 4.00E+00 | 5.92E-03 | 0.903874681 | |
|  | ENSG00000215912 | 1 | TTC34 | 2567415 | rs6666788 | 1 | 2567500 | G | A | 0.690855 | -2.39E-01 | 4.32E-02 | 4.09E-08 | -4.71E-01 | 7.21E-02 | 6.90E-11 | 5.07E-01 | 1.20E-01 | 2.51E-05 | 1.05E-01 | 8.00E+00 | 1.66E-02 | 0.37893345 | |
|  | ENSG00000086504 | 16 | MRPL28 | 417384 | rs3743884 | 16 | 420678 | T | C | 0.523857 | -2.11E-01 | 4.12E-02 | 3.61E-07 | -5.85E-01 | 5.96E-02 | 9.35E-23 | 3.60E-01 | 7.94E-02 | 5.69E-06 | 1.79E-01 | 2.00E+01 | 5.92E-03 | 0.10934633 | |
|  | ENSG00000184110 | 16 | EIF3C | 28699879 | rs62036622 | 16 | 28837203 | G | T | 0.33002 | -1.79E-01 | 3.95E-02 | 6.06E-06 | 6.43E-01 | 1.07E-01 | 1.74E-09 | -2.79E-01 | 7.68E-02 | 2.88E-04 | 3.88E-01 | 2.00E+01 | 4.39E-02 | 0.856427754 | |
|  | ENSG00000259982 | 16 | CDC37P1 | 28711615 | rs11641216 | 16 | 28893532 | G | A | 0.329026 | -1.76E-01 | 3.95E-02 | 9.06E-06 | 7.63E-01 | 9.80E-02 | 7.21E-15 | -2.31E-01 | 5.97E-02 | 1.10E-04 | 7.91E-01 | 2.00E+01 | 5.46E-02 | 0.08643274 | |
| HLA DR on CD14+ CD16- monocyte | ENSG00000271581 | 6 | XXbac-BPG248L24.12 | 31324424 | rs2853951 | 6 | 31236115 | T | C | 0.675944 | -1.50E-01 | 3.08E-02 | 1.13E-06 | 6.24E-01 | 1.06E-01 | 3.53E-09 | -2.41E-01 | 6.40E-02 | 1.70E-04 | 1.55E-02 | 1.20E+01 | 2.59E-02 | 0.03332164 | |
|  | ENSG00000225851 | 6 | HLA-S | 31349851 | rs2524096 | 6 | 31236467 | T | G | 0.451292 | -1.73E-01 | 2.87E-02 | 1.74E-09 | 8.79E-01 | 1.03E-01 | 1.19E-17 | -1.97E-01 | 3.99E-02 | 8.16E-07 | 7.70E-02 | 2.00E+01 | 2.02E-04 | 0.143366853 | |
|  | ENSG00000272221 | 6 | XXbac-BPG181B23.7 | 31362066 | rs28366140 | 6 | 31364288 | G | C | 0.39662 | 1.48E-01 | 2.90E-02 | 3.75E-07 | -1.15E+00 | 1.17E-01 | 1.67E-22 | -1.29E-01 | 2.86E-02 | 6.40E-06 | 2.12E-01 | 2.00E+01 | 1.27E-03 | 0.00024796 | |
|  | ENSG00000198502 | 6 | HLA-DRB5 | 32485120 | rs71549223 | 6 | 32498094 | A | G | 0.83499 | -3.50E-01 | 4.72E-02 | 1.44E-13 | -1.03E+00 | 1.21E-01 | 1.45E-17 | 3.40E-01 | 6.07E-02 | 2.15E-08 | 9.02E-02 | 2.00E+01 | 6.09E-06 | 0.5321688 | |
|  | ENSG00000229391 | 6 | HLA-DRB6 | 32520490 | rs9271367 | 6 | 32586908 | A | G | 0.687873 | -4.71E-01 | 4.08E-02 | 2.83E-30 | 1.05E+00 | 9.45E-02 | 1.75E-28 | -4.50E-01 | 5.63E-02 | 1.37E-15 | 1.13E-01 | 2.00E+01 | 6.76E-13 | 0.2457478 | |
|  | ENSG00000196126 | 6 | HLA-DRB1 | 32546546 | rs74290599 | 6 | 32518785 | T | A | 0.327038 | -8.47E-01 | 4.98E-02 | 1.84E-62 | 8.05E-01 | 9.23E-02 | 2.81E-18 | -1.05E+00 | 1.36E-01 | 8.60E-15 | 8.04E-02 | 2.00E+01 | 3.40E-12 | 0.15684279 | |
| CD4 on CM CD4 + | ENSG00000224796 | 6 | RPL32P1 | 33047228 | rs1126504 | 6 | 33048457 | G | C | 0.299205 | -1.48E-01 | 3.00E-02 | 8.28E-07 | -9.21E-01 | 1.06E-01 | 3.32E-18 | 1.61E-01 | 3.75E-02 | 1.75E-05 | 2.24E-01 | 2.00E+01 | 3.46E-02 | 0.003719088 | |
| CD80 on CD62L+ plasmacytoid DC | ENSG00000173085 | 4 | COQ2 | 84182689 | rs55692463 | 4 | 84149001 | T | C | 0.259443 | -2.61E-01 | 3.03E-02 | 1.05E-17 | 4.20E-01 | 7.62E-02 | 3.42E-08 | -6.22E-01 | 1.34E-01 | 3.34E-06 | NA | NA | 6.61E-03 | 0.00484613 | |
| CD8 on EM CD8br | ENSG00000231259 | 2 | AC125232.1 | 87257800 | rs10205992 | 2 | 87279109 | T | C | 0.516899 | -1.19E-01 | 2.77E-02 | 1.79E-05 | -7.94E-01 | 8.33E-02 | 1.54E-21 | 1.50E-01 | 3.83E-02 | 8.98E-05 | 7.23E-01 | 2.00E+01 | 3.55E-02 | 0.183695334 | |
|  | ENSG00000123737 | 4 | EXOSC9 | 122722472 | rs3828485 | 4 | 122722368 | A | G | 0.342942 | 1.24E-01 | 2.98E-02 | 3.31E-05 | 5.77E-01 | 7.17E-02 | 8.43E-16 | 2.14E-01 | 5.81E-02 | 2.22E-04 | 5.90E-02 | 1.40E+01 | 6.27E-02 | 0.29568363 | |
|  | ENSG00000204642 | 6 | HLA-F | 29690552 | rs2523387 | 6 | 29708090 | A | G | 0.460239 | 2.04E-01 | 2.97E-02 | 7.45E-12 | -3.10E-01 | 4.62E-02 | 1.85E-11 | -6.59E-01 | 1.37E-01 | 1.54E-06 | 2.79E-01 | 2.00E+01 | 1.52E-03 | 0.28468954 | |
|  | ENSG00000230795 | 6 | HLA-K | 29894236 | rs2747467 | 6 | 29659124 | G | A | 0.275348 | -2.47E-01 | 3.70E-02 | 2.69E-11 | -6.66E-01 | 1.04E-01 | 1.47E-10 | 3.71E-01 | 8.02E-02 | 3.71E-06 | 9.03E-02 | 2.00E+01 | 2.44E-03 | 0.356719364 | |
| HLA DR on DC | ENSG00000204644 | 6 | ZFP57 | 29640169 | rs2747431 | 6 | 29648564 | T | C | 0.246521 | 1.81E-01 | 3.98E-02 | 5.70E-06 | 8.74E-01 | 9.92E-02 | 1.26E-18 | 2.07E-01 | 5.12E-02 | 5.34E-05 | 2.47E-01 | 2.00E+01 | 8.81E-03 | 0.02856913 | |
|  | ENSG00000273340 | 6 | MICE | 29709508 | rs9258275 | 6 | 29730504 | G | A | 0.371769 | -1.51E-01 | 3.14E-02 | 1.58E-06 | 7.20E-01 | 9.57E-02 | 5.28E-14 | -2.10E-01 | 5.18E-02 | 5.05E-05 | 1.24E-01 | 2.00E+01 | 8.81E-03 | 0.00384613 | |
|  | ENSG00000235821 | 6 | IFITM4P | 29718506 | rs2394164 | 6 | 29721397 | C | G | 0.27833 | -1.42E-01 | 3.37E-02 | 2.49E-05 | 1.06E+00 | 1.16E-01 | 1.11E-19 | -1.35E-01 | 3.52E-02 | 1.29E-04 | 2.11E-01 | 2.00E+01 | 1.96E-02 | 0.1136853 | |
|  | ENSG00000230795 | 6 | HLA-K | 29894236 | rs2747467 | 6 | 29659124 | G | A | 0.275348 | 1.72E-01 | 3.93E-02 | 1.22E-05 | -6.66E-01 | 1.04E-01 | 1.47E-10 | -2.58E-01 | 7.15E-02 | 2.98E-04 | 6.42E-02 | 2.00E+01 | 3.68E-02 | 0.00581634 | |
|  | ENSG00000271581 | 6 | XXbac-BPG248L24.12 | 31324424 | rs2853951 | 6 | 31236115 | T | C | 0.675944 | -2.10E-01 | 3.38E-02 | 5.70E-10 | 6.24E-01 | 1.06E-01 | 3.53E-09 | -3.37E-01 | 7.88E-02 | 1.85E-05 | 1.21E-02 | 1.20E+01 | 3.65E-03 | 0.11185693 | |
|  | ENSG00000225851 | 6 | HLA-S | 31349851 | rs2524096 | 6 | 31236467 | T | G | 0.451292 | -2.49E-01 | 3.15E-02 | 3.71E-15 | 8.79E-01 | 1.03E-01 | 1.19E-17 | -2.83E-01 | 4.87E-02 | 6.38E-09 | 8.86E-02 | 2.00E+01 | 2.52E-06 | 0.2957634 | |
|  | ENSG00000196126 | 6 | HLA-DRB1 | 32546546 | rs74290599 | 6 | 32518785 | T | A | 0.327038 | -5.91E-01 | 5.56E-02 | 6.32E-26 | 8.05E-01 | 9.23E-02 | 2.81E-18 | -7.35E-01 | 1.09E-01 | 1.56E-11 | 9.12E-02 | 2.00E+01 | 1.03E-08 | 0.567186533 | |
| HLA DR on CD33dim HLA DR+ CD11b+ | ENSG00000196301 | 6 | HLA-DRB9 | 32427598 | rs72844187 | 6 | 32521419 | G | C | 0.368787 | -5.00E-01 | 7.48E-02 | 3.21E-11 | 1.01E+00 | 9.99E-02 | 7.78E-24 | -4.96E-01 | 8.92E-02 | 2.59E-08 | 1.02E-01 | 2.00E+01 | 1.92E-05 | 0.38856835 | |
|  | ENSG00000196126 | 6 | HLA-DRB1 | 32546546 | rs74290599 | 6 | 32518785 | T | A | 0.327038 | -5.13E-01 | 7.14E-02 | 9.92E-13 | 8.05E-01 | 9.23E-02 | 2.81E-18 | -6.38E-01 | 1.15E-01 | 2.91E-08 | 1.17E-01 | 2.00E+01 | 1.92E-05 | 0.7984613 | |
| HLA DR on CD33- HLA DR+ | ENSG00000196126 | 6 | HLA-DRB1 | 32546546 | rs74290599 | 6 | 32518785 | T | A | 0.327038 | -4.15E-01 | 7.15E-02 | 7.58E-09 | 8.05E-01 | 9.23E-02 | 2.81E-18 | -5.16E-01 | 1.07E-01 | 1.34E-06 | 9.43E-02 | 2.00E+01 | 6.61E-04 | 0.67894613 | |
|  | ENSG00000235821 | 6 | IFITM4P | 29718506 | rs2394164 | 6 | 29721397 | C | G | 0.27833 | -1.74E-01 | 4.24E-02 | 4.41E-05 | 1.06E+00 | 1.16E-01 | 1.11E-19 | -1.64E-01 | 4.40E-02 | 1.89E-04 | 9.15E-02 | 2.00E+01 | 4.72E-02 | 0.2083775 | |
| GWAS: genome-wide association study.  eQTL: expression quantitative trait loci.  SMR: summary-data-based Mendelian randomization.  HEIDI: heterogeneity in dependent instruments. Only genome-wide significant eQTLs (P<5E-8) are taken into the analysis. We report SNP-gene combinations with PSMR < genome-wide significance Benjamin Hochberg correction threshold of PFDR＜0.05, and survived after the heterogeneity test (PHEIDI＞0.05). β in GWAS association, regression coefficient of cancer on SNP, log(OR).  SE, standard error.  β in eQTL association, regression coefficient of gene expression on SNP.  β in SMR association, regression coefficient of cancer on gene expression. | | | | | | | | | | | | | | | | | | | | | | | |  |
